# Can a standards-based approach improve access to and quality of primary health care? Findings from an end-of-project evaluation in Ghana

**DOI:** 10.1371/journal.pone.0216589

**Published:** 2019-05-10

**Authors:** Christina Maly, Richard Okyere Boadu, Carina Rosado, Aliza Lailari, Bernard Vikpeh-Lartey, Chantelle Allen

**Affiliations:** 1 Monitoring, Evaluation and Research, Jhpiego, Johns Hopkins University Affiliate, Baltimore, MD, United States of America; 2 Department of Health Information Management, University of Cape Coast, Cape Coast, Ghana; 3 Strategy & Analytics, Deloitte Consulting, LLP, Rossylyn, VA, United States of America; 4 Strategic Information and Evaluation, Elizabeth Glaser Pediatric AIDS Foundation, Washington, DC, United States of America; 5 Independent Development Consultant, Takoradi, Ghana; 6 Technical Leadership and Innovations, Jhpiego, Johns Hopkins University Affiliate, Baltimore, MD, United States of America; Public Health Foundation of India, INDIA

## Abstract

**Background:**

Jhpiego implemented a 5-year project to strengthen the Community-Based Health Planning and Services (CHPS) model in six coastal districts of Ghana’s Western Region. The project utilized a quality improvement approach (Standards-Based Management and Recognition [SBM-R]) to strengthen implementation fidelity of the CHPS model. This article presents findings from an end-of-project evaluation comparing quality, access to care, and experience of care in intervention and comparison CHPS zones.

**Methods:**

A non-equivalent, posttest–only, end-of-project evaluation compared 12 randomly selected intervention zones with 12 matched comparison zones. Data from standards-based assessments measured provision of care in three categories: community engagement, clinical services, and facility readiness and management. Access to and experience of care were assessed using a household survey of 426 randomly selected community members from the selected CHPS zones. Bivariate and multivariate analyses were conducted to compare performance on these measures between intervention and comparison CHPS zones.

**Results:**

Overall, intervention zones outperformed comparison zones on achievement of standards (83.6% vs 58.8%) across all three assessment categories, with strongest results in community engagement (85.7% vs. 41.4%). Respondents in intervention zones were more than twice as likely to have received a home visit from a community health officer, three times as likely to have a home visit from a community health volunteer, and more likely to have attended a health talk (41.9% vs. 27.0%). Client experiences of care were reported as positive in both study arms.

**Conclusions:**

The evaluation demonstrated improved access to quality care; however, there were very few differences in client experience of care between intervention and comparison zones. As Ghana and other countries are committed to scaling up universal health care, a pragmatic approach such as SBM-R could prove useful to engage both facility- and community-based service providers, as well as community members, to improve provision of care.

## Introduction

Ghana has passed several health-related laws in line with global and/or regional priorities [1] and introduced a national health insurance scheme, while moving out of low-income status as a country in 2010 [2]. In addition, the crude maternal mortality ratio has dropped by nearly 50% over the last 25 years (634 per 100,000 live births in 1990 to 319 in 2015) [3] and over 90% of women attend at least one antenatal care visit [4]. Despite these improvements, Ghana made only slow progress on Millennium Development Goals 4 and 5, reducing under-5 mortality and maternal mortality, respectively [5], and significant inequities in income and health outcomes remain [1,6,7]. To address these challenges, Ghana has prioritized universal provision of primary health care in line with the Ouagadougou Declaration on Primary Health Care and Health Systems in Africa [8] and Sustainable Development Goal 3, which includes provision of universal health coverage (UHC) [9]. To operationalize this goal at the national level, Ghana began scaling up Community-Based Health Planning and Services (CHPS) [10]. The CHPS model delivers health care directly to the household and community levels by placing community health officers (CHOs) in communities and using community-based approaches for delivery of primary health services.

Sustainable Development Goal 3 is focused on universal access to care, and also on high-quality care; thus, as Ghana and other countries strive for UHC, an emphasis on quality of care is needed [11–13]. As coverage of care expands, greater understanding of the quality of health services will be required, both in terms of the provision of care and the client experience of care [13–15].

A good example of the increasing global commitment to quality of care and valuing the client’s experience of care is the World Health Organization’s new quality of care framework for maternal and newborn health [16]. The framework articulates the importance of provision of care and client experience of care within the health systems building blocks. Ensuring the quality of care through this framework may include implementation of quality improvement initiatives with increased monitoring of progress, as well as engagement and feedback on clients’ experiences of care [16].

Audit and feedback have been shown to improve quality of care [17]. In this approach, an individual’s or facility’s practice or performance is compared to professional standards or targets (audited), and the results are fed back to the individual or facility. The aim of this process is to engage health care providers in identifying and addressing gaps to achieve the required professional standards. This approach formed the cornerstone of the Supportive Technical Assistance for Revitalizing CHPS (STAR CHPS) project that was initiated in 2011 in six coastal districts of Western Region, Ghana. This paper presents results of an end-of-project evaluation in 2015 that assessed access to and quality of community-based primary health services provided in STAR CHPS-supported sites, compared to non-intervention sites.

### The CHPS model

Based on the positive findings of operations research conducted by the Navrongo Health Research Center in the Upper East Region between 1994 and 1996, the Ghana Ministry of Health (MOH) launched CHPS in 1999 as a national health policy initiative [18]. The Ghana Health Services (GHS) mobilized community support for CHOs posted to a CHPS zone to deliver primary health care at the household and community levels to improve geographic access. A CHPS zone encompasses up to 5,000 persons or 750 households and is usually comprised of several communities [18]. The community participates in CHPS through an elected community health management committee (CHMC) and by nominating or serving as community health volunteers (CHVs) in their CHPS zone. CHPS is an important strategy to improve geographic access to primary health care and maternal and child health services, particularly for remote and rural populations [19]. Table 1 describes the roles and responsibilities of the various members of the CHPS model outlined in the national guidelines [20].

**Table 1:** Members of the CHPS model.

Community Health Officers (CHOs)	A community health nurse, enrolled nurse, or midwife trained in the CHPS model and placed in a CHPS zone to work with communities to provide basic primary health care.
Community Health Management Committees (CHMCs)	Community members elected by chiefs and community opinion leaders. CHMCs serve as the primary liaison between community members and CHOs and are responsible for the welfare of CHOs. CHMCs also select and supervise CHVs.
Community Health Volunteers (CHVs)	Laypersons in the communities elected by chiefs, elders, and CHMC members at a formal community forum, or *durbar*. CHVs support the CHO by providing health education and conducting community outreach and home visits where they provide health education and referrals and/or treat minor ailments. They receive technical supervision from the CHO and management oversight from the CHMC.
District Health Management Team (DHMT)	District-level health managers (e.g., district health directors, public health nurses, health information officers) who work together to provide technical leadership, management, and expertise to ensure delivery of facility- and community-based health services in the district.

Ghana has demonstrated a national commitment to implementation and scale-up of the CHPS strategy by investing in infrastructure, creating and expanding a cadre of community health nurses through community health nursing schools, and re-aligning CHPS districts to electoral areas [20]. However, CHPS scale-up has been slower than anticipated. In 2013, when the STAR CHPS project was being designed, only about 22% of CHPS zones were reported by the MOH as being functional [21]. Nyonator [18] labeled this the CHPS “implementation gap.”

### Project description

**Fig 1:**
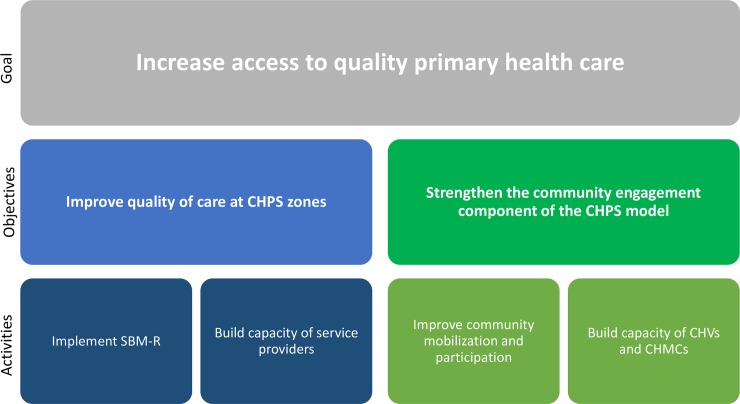
STAR CHPS project design.

In 2011, Jhpiego received a 5-year grant from the Jubilee Partners (Tullow Oil, Kosmos Energy, Anadarko Petroleum, Ghana National Petroleum Corporation, and Petro SA) to implement the STAR CHPS project. The project partnered with the GHS Western Regional Health Directorate to support implementation and scale-up of CHPS activities in 62 CHPS zones across all six coastal districts of this region. The goal was to increase access to quality primary health care (Fig 1) by improving the quality of services and strengthening community engagement. The STAR CHPS project was designed to close the “implementation gap” by improving fidelity to the national CHPS model, especially community engagement and support components. The project built the capacity of all members of the CHPS team to support implementation and service delivery, and utilized a quality improvement approach to strengthen fidelity to the CHPS model and to ensure that all implementation steps and milestones of the model were achieved [20]. The assumption underlying the project’s approach was that improving quality of care would also improve client experience of care, and improving fidelity to the CHPS model would result in a greater number of clients having access to primary care [15].

#### Improving quality of care

This project applied the Standards-Based Management and Recognition (SBM-R) audit and feedback quality improvement approach [17]. SBM-R uses continuous audit, action planning, feedback, and recognition of progress to improve service quality. It engages health service providers and managers to work together to address gaps in performance. The theoretical foundation of the SBM-R approach is fully described by Necochea and colleagues [22].

Traditionally, this approach has been used in facility-based settings; this was the first time SBM-R was applied to a community-based model. STAR CHPS and GHS staff jointly implemented SBM-R with CHOs, CHMCs, CHVs, and DHMTs.

Over the 5-year life of the project (2011–2016), the following four-step process was followed: 1) set standards—set 188 standards with 1,242 verification criteria based on an extensive desk review of national guidance and strategies to consolidate expected clinical services, facility readiness and management, and community engagement for CHPS; 2) implement standards—conducted assessments, action planning, support visits, and capacity development; 3) measure progress—re-assessed; and 4) provide recognition—provided reward and recognition events and certificates [23].

#### Building capacity of health service providers

To address the specific service provider capacity gaps identified by the initial SBM-R assessments, the STAR CHPS and GHS staff delivered a modified version of the GHS-recommended 12-day course for CHOs that included orientation on the CHPS model, community engagement, and specified clinical services. After training and assessments, GHS and STAR CHPS conducted supportive supervision visits to follow up on progress and support CHOs to address performance issues that had been identified. In addition, the project and GHS facilitated monthly district-based CHOs meetings to share progress and lessons learned.

#### Strengthening community mobilization and participation and building capacity of CHMCs and CHVs

STAR CHPS supported each of the DHMTs to work with the chiefs and community opinion leaders to establish or strengthen the community-related structures, including identification and support of CHVs and establishment of CHMCs in each CHPS zone, in accordance with national policy and implementation guidance.

Once CHVs and CHMCs were selected and formally introduced to their communities, the project oriented them on the CHPS model and various tools and approaches to be used in their work. CHVs learned how to conduct home visits in keeping with the GHS national policy and STAR CHPS provided home visiting bags to all CHVs with simple supplies and equipment.

## Methods

A posttest only, non-equivalent control group design was adapted for the formal evaluation at the end of the project. Comparison groups were selected from districts adjacent to the six project districts within the same region.

The project encompassed 62 CHPS zones. Nineteen CHPS zones that had received less than 2 years of project support and five urban CHPS zones were excluded from the sampling pool. The remaining 38 zones were stratified by duration of project support (3 or 4 years), and 12 were randomly sampled to ensure proportional representation by duration of support. Frequency matching on the following three criteria was used to randomly sample an additional 12 comparison CHPS zones from similar districts in the same region: 1) number of years of existence; 2) staff-to-population ratio; and 3) existence of a brick-and-mortar facility, as the CHPS model can be implemented with or without a physical facility. However, for the purposes of this evaluation we only included those with a physical structure.

A household survey was conducted for the purpose of gathering data on access to and perceptions of quality of care provided through CHPS zones. For this survey, three communities from each of the 24 sampled CHPS zones were selected. The community in which the CHPS zone facility was located was purposively selected and two other communities within the catchment area were randomly selected. Within each zone, 18 households were surveyed across the three selected communities, for a maximum total sample size of 432. The sample size was calculated to detect a 15% absolute difference in the proportion of survey respondents who reported receiving high-quality care at intervention zones versus comparison zones with 80% power and the type 1 error fixed at 5%. To account for possible confounding due to similarities among clients being served by the providers from any one CHPS zone, within-facility correlation of responses was assumed to be 0.01 based on routine monitoring data from similar projects. Data were collected in the 24 sampled CHPS zones over 3 weeks in October and November 2015. Please see S2 Tool Household Survey for full details.

### SBM-R assessment tool, data collection, and analysis

The SBM-R portion of the study included all members of the CHPS model—CHOs, CHVs, CHMC members, and DHMTs. Current or retired GHS staff conducted the SBM-R assessments after participating in a 4-day workshop. Training topics included research ethics and informed consent, standard study operating procedures, standardized observations and scoring for the SBM-R assessment tool, and use of a tablet-based application for SBM-R assessment data collection. Thirteen of the 15 data collectors had previously conducted SBM-R assessments for routine project activities. GHS staff were assigned to collect data in CHPS zones where they had not previously worked or done assessments for the project.

The SBM-R assessment tool, which was the same tool used during routine project assessments, covered 44 areas comprised of 188 performance standards and 1,242 verification criteria (Table 2) across all four groups of participants. For the purpose of this evaluation, the criteria were organized into three thematic categories: 1) community engagement; 2) facility readiness and management; and 3) clinical services [24]. Interviews, role plays, and observations of patient care and other service provision were used to determine if each verification criteria should be scored “yes” or “no.” When a CHMC did not exist or there was no CHV in a CHPS zone, the assessors marked the criterion as “no.” Overall scores for each CHPS zone were calculated as the percentage of verification criteria scored “yes.” Scores for each of the three thematic categories were similarly calculated (Table 3). Table 4 presents percentage of verification criteria achieved for each of the community engagement assessment areas by study arm. Please see S1 Tool SBM-R for full details.

**Table 2:** Sample characteristics of facilities included in SBM-R assessment and household questionnaire, by study arm.

Sample Characteristics	CHPS Zones	
	**Intervention** **(n = 12)**	**Comparison** **(n = 12)**
**CHPS Zone Characteristics** Mean (Standard Deviation)		
Staff-to-population ratio	1: 1,920 (636)	1: 1,588 (830)
Years in existence	5.4 (2.9)	5.3 (3.1)
**SBM-R Assessment Respondents** Mean (Standard Deviation)		
Number of SBM-R participants per CHPS zone	4.3 (0.65)	3.8 (1.0)
Percentage of CHOs sampled for SBM-R data collection	86% (37)	73% (23)
** **	**Intervention** **(n = 215)**	**Comparison** **(n = 211)**
**Household Questionnaire Respondents**		
**Sex**		
Male	35.8%	28.4%
Female	64.2%	71.6%
**Age** Mean (Range)	38.8 (18–87)	39.9 (18–85)
18–24	18.1%	11.4%
25–29	15.8%	10.9%
30–39	23.3%	33.2%
40–49	17.2%	23.3%
50 and older	25.5%	21.3%
**Marital status**		
Married or living together	77.2%	82.5%
Divorced, separated or widowed	13.5%	13.3%
Never married or living together	8.8%	3.8%
No response	0.5%	0.5%
**Number of living children** Mean (Range)	4.0 (0–15)	4.2 (0–14)
0	9.8%	4.7%
1–2	21.4%	25.6%
3–4	27.4%	25.6%
5–6	25.1%	27.0%
7 and above	16.3%	17%
**Education**		
None/non formal	27.4%	26.5%
Primary	25.6%	23.7%
Junior	35.3%	40.3%
Senior, tertiary, and higher	11.6%	9.5%
**Religion**		
Christian	82.8%	82.5%
Muslim	7.0%	11.9%
Traditional/spiritualist/no religion/other	10.2%	4.7%
No response	0.0%	1.0%
**Household assets score**^**a**^ Mean (range)	4.67 (0–10)	5.25 (0–10)
	**Intervention** **(n = 139)**	**Comparison** **(n = 150)**
**Time to CHPS facility among respondents who report any CHPS facility visits**		
29 minutes or less	48.3%	59.5%
30 minutes or more	46.3%	32.5%
No response	5.4%	8.0%

CHPS, Community-Based Health Planning and Services; SBM-R, Standards-Based Management and Recognition; CHO, community health officer.

^a^ Household assets were an additive score with a potential range of 0 to 10 and included various household materials and whether respondents use a shared toilet

**Table 3:** SBM-R assessment: percentage of verification criteria achieved by category and study group.

	Mean % of Verification Criteria Achieved (Standard Deviation)		Adjusted Analysis^a^		
	Intervention (n = 12)	Comparison (n = 12)	Risk Ratio	95% CI	P-Value
**Community Engagement (330 criteria)**	85.7% (8.9)	41.4% (11.0)	2.00	1.72–2.33	<0.001
CHO (150 criteria)	92.1% (10.0)	57.5% (16.4)	1.54	1.34–1.77	<0.001
CHV (114 criteria)	83.0% (11.0)	26.9% (12.6)	2.96	2.67–3.29	<0.001
CHMC (66 criteria)	75.9% (18.1)	29.7% (18.4)	2.52	1.44–4.42	0.001
**Facility Readiness & Management (269 criteria**^**b**^**)**	65.8% (20.5)	50.8% (13.7)	1.27	1.12–1.45	<0.001
**Clinical Services (643 criteria)**	90.0% (8.9)	71.1% (13.5)	1.25	1.10–1.41	0.001
**Overall (1,242 criteria)**	83.6% (9.1)	58.8% (9.6)	1.39	1.24–1.57	<0.001

CHO, community health officer; CHV, community health volunteer; CHMC, community health management committee; CI, confidence interval.

^a^ Multivariate model controlled for facility maturity, staff-to-population ratio, and clustering at the district level.

^b^ For pragmatic purposes readiness (e.g. infrastructure, equipment, medicines) and management (e.g., data collection, reporting and use; financial and logistics management) were grouped together and a summary score was calculated

**Table 4:** Multivariate analyses of percentage of verification criteria achieved for community engagement assessment areas.

	Mean % of Verification Criteria Achieved (Standard Deviation)		Adjusted Analysis^a^		
	Intervention (n = 12)	Comparison (n = 12)	Risk Ratio	95% CI	P-Value
**Community Engagement**					
*CHO*					
Health promotion and health education	87.4% (22.0)	49.3% (25.8)	1.62	1.2–2.19	0.002
Disease surveillance	92.7% (10.2)	70.5% (20.6)	1.25	1.1–1.42	<0.001
Home visits	92.8% (18.2)	60.9% (26.8)	1.44	1.2–1.80	0.001
School health	93.4% (56.8)	79.0% (40.9)	1.15	1.0–1.29	0.016
Outreach activities	96.6% (5.6)	52.8% (39.5)	1.67	1.1–2.49	0.012
Supporting CHVs	75.0% (38.4)	2.1% (7.2)	37.32	6.4–219.07	<0.001
Working with the CHMC	94.0% (8.9)	22.7% (33.4)	4.37	2.3–8.49	<0.001
*CHV*					
Disease prevention and environmental sanitation	91.7% (14.4)	28.1% (24.5)	3.12	1.6–6.0	0.001
Home visiting (procedures)	91.3% (10.7)	47.7% (26.8)	1.90	1.6–2.3	<0.001
Home management of minor ailments	91.1% (10.6)	23.2% (20.0)	3.93	2.5–6.3	<0.001
Community outreach	95.5% (6.5)	46.9% (24.4)	1.97	1.6–2.4	<0.001
Logistics (supplies and equipment)	63.5% (25.7)	0.4% (1.0)	132.72	47.2–373.0	<0.001
*CHMC*					
Governance, membership, and operation	92.9% (9.1)	44.5% (27.7)	2.01	1.2–3.4	0.01
Selection and supervision of community volunteers	77.1% (18.3)	11.4% (15.8)	1.97	1.0–3.8	<0.001
Welfare of CHO (include security)	77.4% (21.5)	39.3% (35.1)	1.97	1.0–3.8	0.037
Facility and service maintenance	81.0% (26.1)	34.%5 (28.9)	2.25	1.3–4.0	0.005
Resource mobilization and management	39.5% (49)	4.4% (15.4)	12.04	1.4–101.4	0.022

CHO, community health officer; CHV, community health volunteers; CHMC, community health management committee; CI, confidence interval.

^a^ Multivariate model controlled for facility maturity, staff-to-population ratio, and clustering at the district level.

Means for staff-to-population ratio, number of CHOs per CHPS zone, and years in existence were calculated for intervention and comparison zones from data obtained from the Regional Health Directorate. To find the proportion of CHOs sampled, the percentage of CHOs sampled per CHPS zone was first calculated, and then the average for intervention and comparison CHPS zones overall was calculated. For both the bivariate and multivariate analyses, we used a generalized linear regression model with Poisson distribution. The multivariate analyses included the matching criteria, facility maturity, and staff-to-population ratio as covariates in the model. Analyses were completed using Stata 13.

### Household survey tool, data collection, and analysis

A team of 15 experienced household survey data collectors participated in a 4-day training workshop on the objectives of the study, research ethics, including obtaining informed consent, study standard operating procedures, and use of the mobile data collection application, CommCare (www.commcarehq.org). The household questionnaire was developed by the project team for the specific purpose of the end-line evaluation (S2 Tool). The survey was informed by a literature review of other studies that examined access to and quality of care [25–26]. After obtaining informed written consent, data collectors administered the 62-question household survey to community members living within sampled CHPS zones.

The data collectors worked with local leaders in each community to identify the center of the community where a random direction was chosen using the spin-the-bottle technique. A random number application was used to determine the first household to approach, after which every third house in the same direction was approached. If the community border was reached, the data collector would turn clockwise and continue visiting the third nearest house using the same method. This process continued until the target number of households in the community was reached. If multiple households lived in the same house, a random number was used to select the household to screen, then a recruitment script was used to identify the person in the household most likely to accompany a family member to a health facility. Households that included a potential SBM-R assessment respondent were excluded, as were those under the age of 18 or those who had lived in the community less than 1 year.

Respondents were asked about various aspects of their experiences of care for both facility and home visits: amount of time spent, perceived knowledge and skills of providers, respect of clients, confidentiality, and overall satisfaction. As a proxy for economic status, information on household assets was collected and used to create a composite household assets score. This was an additive score with a potential range of 0 to 10, based on household ownership of various household materials and whether respondents used a shared toilet. As the GHS mobilized CHOs at CHPS zones to deliver primary health care at the household and community levels to improve geographic access, respondents were also asked about their number of facility visits, receipt of home visits, and participation in community-based activities as a proxy for access to primary health care. Readiness included infrastructure, medicines, and tests required to deliver the basic package of clinical services in line with the WHO SARA that outlines the prerequisite inputs for quality services [24]. Descriptive statistics and bivariate analysis were conducted to determine whether there were significant differences between intervention and comparison zones on a set of variables after adjusting for clustering at the household and community levels, using the combined weights in Stata survey commands to account for the probability of selection into the intervention zone (Table 5).

**Table 5:** Bivariate analyses of household questionnaire by study arm.

	Percent Reported by Study Arm		
	Intervention	Comparison	P-value
**Access to Services**			
**Number of CHPS facility visits reported**	**(n = 215)**	**(n = 211)**	0.12
1 or more visits	68.4%	77.3%	
0	31.2%	22.3%	
No response	0.5%	0.5%	
**Number of CHO home visits reported**	(n = 215)	(n = 211)	<0.001
1 or more visits	39.5%	18.0%	
0	60.5%	82.0%	
**Number of CHV home visits reported**	**(n = 215)**	**(n = 211)**	<0.001
1 or more visits	29.8%	8.5%	
0	68.8%	91.5%	
No response	1.4%	0.0%	
**Attended a health talk**			0.004
Yes	41.9%	27.0%	
No	58.1%	72.5%	
No response	0.0%	0.5%	
**Attended a child welfare clinic**			0.249
Yes	55.7%	62.7%	
No	42.8%	36.8%	
No response	1.5%	0.5%	
**Client Experience of Care**			
***CHPS Facility Visit***	**(n = 147)**	**(n = 163)**	
**Right amount of time spent**			0.049
The right amount of time	78.9%	71.2%	
Too long	12.2%	16.6%	
Too short	4.1%	10.4%	
No response	4.8%	1.8%	
**Knowledge and skills to perform services**			0.009
Strongly agree or somewhat agree	89.1%	96.9%	
Strongly disagree or somewhat disagree	4.1%	2.5%	
No response	6.8%	0.6%	
**Respectful of patients**			0.281
Respectful	53.1%	46.6%	
Somewhat respectful or not respectful at all respectful	46.3%	53.4%	
No response	0.7%	0.0%	
**Keep health and personal information private**			0.047
Strongly agree or somewhat agree	84.4%	92.6%	
Strongly disagree or somewhat disagree	4.1%	3.1%	
No response	11.6%	4.3%	
**CHPS facility visit—client overall satisfaction**			0.721
Somewhat satisfied or very satisfied	92.5%	91.4%	
Somewhat dissatisfied or very dissatisfied	7.5%	8.6%	
***CHO Home Visit***	**(n = 64)**	**(n = 34)**	
**Right amount of time spent**			0.053
The right amount of time	84.4%	64.7%	
Too long	9.4%	14.7%	
Too short	4.7%	20.6%	
No response	1.6%	0.0%	
**Knowledge and skills to perform services**			0.464
Strongly agree or somewhat agree	98.4%	100.0%	
No response	1.6%	0.0%	
**Respectful when visiting home**			0.251
Agree	71.9%	82.4%	
Somewhat agree, somewhat disagree, strongly disagree	28.1%	17.6%	
No response			
**Keep health and personal information private**			N/A
Strongly agree or somewhat agree	96.9%	100.0%	
No response	3.1%	0.0%	
**CHO home visit—client glad for the visit**			N/A
Strongly agree or somewhat agree	100.0%	100.0%	
***CHV Home Visit***	**(n = 60)**	**(n = 18)**	
**Right amount of time spent**			0.06
The right amount of time	88.5%	72.2%	
Too long	6.6%	5.6%	
Too short	3.3%	22.2%	
No response	1.6%	0.0%	
**Knowledge and skills to perform services**			0.11
Strongly agree or somewhat agree	91.7%	88.9%	
Strongly disagree or somewhat disagree	1.7%	11.1%	
No response	6.7%	0.0%	
**Respectful when visiting home**			0.395
Agree	71.7%	61.1%	
Somewhat agree, somewhat disagree, strongly disagree	28.3%	38.9%	
**CHV home visit—client glad for the visit**			0.139
Strongly agree or somewhat agree	96.7%	94.4%	
Strongly disagree or somewhat disagree	0.0%	5.6%	
No response	3.3%	0.0%	

CHPS, Community-Based Health Planning and Services; CHO, community health officer; CHV, community health volunteers.

Ethical approval for the study was obtained from Johns Hopkins Bloomberg School of Public Health Institutional Review Board (00006456) and the Ghana Health Service Ethical Review Committee (GHS-ERC 10/09/15).

## Results

### Background characteristics

CHPS zones in the intervention and comparison arms were similar in staff-to-population ratio and years in existence (Table 2). An average of 3 CHOs worked at each CHPS zone (range of 1 to 4), except for one comparison zone that had 8 CHOs. A total of 137 individuals responded to the SBM-R assessments across the 24 CHPS zones—53 CHOs, 23 CHVs, 22 CHMC members, and 39 DHMT members. An average of 4.1 people per CHPS zone participated in the study (intervention: 4.3; comparison: 3.8). DHMT members were not included in the average as they support more than one CHPS zone. The slightly higher average in the intervention zones was due in part because two comparison CHPS zones did not have a CHMC and one of those zones was also lacking a CHV. All of these findings are presented in Table 2.

A total of 426 individuals responded to the household survey (of 428 screened and consented). On average, the respondents were around 39 years old and had four living children. Most respondents were female, married, and had some formal education. In intervention zones, respondents had lower household asset scores, lived further from a CHPS facility and were less likely to identify as Muslim. Among respondents who reported any CHPS facility visits, those living in intervention zones were generally further away, with less than half (48.3%) reporting being within 30 minutes to the facility, while in comparison zones it was 59.5%. In intervention zones, there were fewer Muslims and more respondents identifying as having traditional, spiritualist, or no religious beliefs. These details are presented in Table 2.

### SBM-R assessments

On the SBM-R assessments, intervention zones significantly outperformed comparison zones on achievement of overall standards-based verification criteria (intervention: 83.6%, comparison: 58.8%, p-value <0.001) (Table 3). The greatest differences in performance were observed in the community engagement assessments (85.7% vs. 41.4%, p-value <0.001), although differences were also seen in the other two categories: clinical services—90.0% vs. 71.1% (p-value <0.001) and facility readiness and management– 65.8% vs. 50.8% (p-value <0.001). As part of the intervention, minor logistic support was provided to a few of the facilities, which may have contributed to some of the differences seen in readiness scores. Cadre-specific analysis of the community engagement assessments shows CHOs in intervention zones achieved 92.1% of verification criteria compared to 57.5% in comparison zones (p-value <0.001). CHVs in intervention zones similarly outperformed those in comparison zones (83.0% vs. 26.9%, p-value, <0.001). CHMCs in interventions zones achieved 75.9% of criteria, whereas only 29.7% of criteria were achieved in comparison zones (p-value <0.001). Findings are presented in Table 3.

Performance was further analyzed by assessment area for each of the three categories. Results for the community engagement assessment areas are presented in Table 4 by cadre of respondent. Within the community engagement category, intervention zones significantly outperformed comparison zones in every assessment area. Intervention zones generally outperformed comparison zones in the other two categories as well. Full results are presented in S1 Table. Within the facility readiness and management area intervention zones achieved 70.5% of verification criteria for financial management, as compared to 47.6% in comparison zones. In terms of clinical services, again the intervention zones fared better than comparison zones. Intervention CHPS zones achieved 95% of verification criteria on child immunization, compared to only 66% of intervention zones.

CHOs in intervention zones were significantly more likely to engage other cadres of the CHPS model versus those in comparison zones. For example, CHOs in intervention zones were 37 times more likely to support CHVs (adjusted risk ratio [ARR] 37.32, p-value <0.001, 95% CI 6.4–219.07) and four times more likely to work with CHMCs (p-value <0.001, 95% CI 2.3–8.49) than their counterparts in comparison zones. Similarly, CHVs were nearly four times more likely (p-value <0.001, 95% CI 2.5–6.3) to be able to manage minor home ailments than those in comparison zones. CHMCs were 12times more likely to outperform comparison zone committees in terms of resource mobilization and management (p-value <0.022, 95% CI 1.4–101.4). For all other assessment areas, intervention CHMCs were approximately twice as likely to outperform comparison zone committees.

### Household survey

The number of CHPS facility visits reported in the year prior to the survey did not differ significantly between respondents in intervention zones and comparison zones, while analysis on items more directly related to the community-based components of the CHPS model did show differences (Table 5). Nearly 40% of respondents in intervention zones reported having at least one home visit from a CHO in the prior year, compared to 18% of respondents in comparison zones (p-value, <0.001). Reports of home visits in the past year from CHVs were more than three times higher in intervention zones (29.8% vs. 8.5%, p-value <0.001). In the year prior to the survey, nearly 42% of respondents in intervention zones attended a health talk led by a CHO, compared to 27% of respondents in comparison zones (p-value <0.004). Attendance at a child welfare clinic did not differ significantly between study arms. While differences in home visits, a proxy for access to services, did demonstrate some differences between study arms, the effect on the client experience of care did not show much variance. Overall satisfaction with visits to CHPS facilities and being appreciative of home visits was overwhelmingly high by respondents in both intervention and comparison zones.

Few differences between the study arms were noted on more specific aspects of client experience of care. Regarding CHOs having the knowledge and skills to perform services during facility visits, there was a difference in overall distribution of responses by study arm (p-value = 0.009). Fewer respondents in intervention versus comparison zones reported that they felt the CHOs had the knowledge and skills to perform services (89.1% vs. 96.9%). However, when asked the same question about interactions with the same cadre of providers during home visits, the responses in the intervention and comparison zones were similar (98.4% vs. 100%). Similarly, there was a statistically significant difference in distribution of responses regarding CHOs keeping client health and personal information confidential during facility visits between study arms (p-value = 0.047). Fewer respondents in intervention versus comparison zones felt CHOs kept their health and personal information confidential during facility visits (84.4% vs. 92.6%), but on the same variable during home visits, responses were similar across study arms.

Respondents in both study arms reported feeling CHOs were more respectful during home visits than during facility visits; with no statistical differences in overall distribution of responses between study arms in either visit setting. When asked about the amount of time spent with a CHO or CHV, respondents in intervention zones were more likely to report the “right amount of time” was spent, rather than the visit being too long or short. The distribution of responses regarding the length of visit between study arms was statistically significant for facility visits (p-value = 0.049). A higher proportion of respondents in the intervention arm reported the facility visit being the “right amount of time” (78.9% vs. 71.2%). For home visits conducted by CHOs, the difference in response distribution (p-value = 0.053) was at, but not below the set value for statistical significance. For CHV home visits, more respondents in intervention zones (88.5%) reported the right amount of time was spent, compared to comparison zones (72.2%). The difference in overall response distribution (p-value = 0.06) did not meet the set value for statistical significance.

## Discussion

Ghana is making progress in achieving Sustainable Development Goal 3 and UHC primarily through the national scale-up and implementation of the CHPS model [27–28]. For these goals to be achieved, services need to be high quality and people-centered within health systems that can respond to local realities, demographics, and resource allocation [29–30]. This evaluation examined access to, and quality of, CHPS services in intervention and comparison sites after 2 to 4 years of project support.

### Access

The CHPS model relies on community engagement as a mechanism to overcome barriers to accessing health care in Ghana by taking health care directly to the homes of community members through home visits, outreach activities, and direct participation of community members. Yet community engagement has been the weakest component of the implementation [31]. As a proxy for access to primary health care, the evaluation asked respondents about visits to CHPS facilities, home visits received, and participation in community-based activities [32]. Intervention zones achieved a significantly higher proportion of standards relating to community engagement than comparison zones and respondents in intervention zones were more likely to have had at least one home visit from a CHV and a CHO and to have attended a community outreach event. These findings suggest this intervention was successful in overcoming one of the biggest hurdles to successful scale-up of the CHPS model. They are in line with findings of Sakeah et al. (2014) who documented the crucial role strengthening community engagement had on increased access to skilled delivery in CHPS zones [33]. Despite intervention zones reaching more than double the percentage of respondents with CHO home visits (39% vs 18%) than comparison zones, this still falls short of the ambitious goals of reaching all households. Given the criticality of home visits as a component of the CHPS model, designing interventions or support that can overcome the challenges related to this component warrants further exploration.

Interestingly, respondents in both intervention and comparison sites reported similar numbers of facility visits. The project team did anticipate that strengthened community engagement and linkages would lead to more confidence and greater utilization of fixed facilities. It may be of interest to explore this result further.

### Quality of care

Various models of quality have been explored and defined in the literature, our evaluation examined two aspects of quality of care: provision of care and client experience of care.

#### Provision of care

A Cochrane review found that an audit and feedback approach is most likely to lead to improvements in practices when baseline performance is low and when the approach is performed by a colleague or supervisor, is provided both verbally and in writing, is repeated over time, and includes targets and an action plan [17]—all aspects present in the STAR CHPS intervention. Consistent with the findings of this review, intervention zones outperformed comparison zones on all three categories of standards—clinical services, facility readiness and management, and, most remarkably, community engagement. This approach was effective in addressing gaps as the standards assisted implementation though articulating not only what to do, but how to do it (i.e., standards with detailed verification criteria). The SBM-R approach used in this project has been implemented in over 30 countries, but is usually focused on delivery of services in a facility [22,34,35]. This project and its evaluation demonstrated the applicability of the approach for a community-based, primary health care service delivery platform and for volunteers and community committees. The performance standards developed in this work were reviewed and referenced by the Ghana MOH during the 2015 update of the CHPs policy because they provided a full description of the details on CHPS service delivery. In addition, they were also included in the 2016 updated CHPS implementation guidance as part of the basic package of services.

#### Client experience of care

The classical Donabedian framework that categorizes dimensions of care into structure, process, and outcomes [36] is frequently the basis for assessing provision and experience of care. Despite the rich legacy of this framework, client experience of care and satisfaction remain challenging to accurately measure and interpret [35,37–39].

In a systematic review of determinants of women’s satisfaction with maternal health care in developing countries, Srivastava et al. (2015) noted that in most interventions, the underlying theory of change proposes that responsive and culturally appropriate care will enhance utilization and thus improve outcomes [15]. However, the review also found that satisfaction ratings were high across almost all studies. They question if this could be due to lack of awareness and low levels of literacy. If poor women have limited access to public services, they may not have a sense of entitlement to health care. Thus, even basic facilities would be satisfactory to them. Consistent with this, our findings showed that respondents in both intervention and the comparison CHPS zones reported very high overall levels of satisfaction with care received in facilities and during home visits.

Despite intervention zones statistically outperforming comparison zones overall on standards-based assessments, respondents in both study arms almost universally reported being satisfied or very satisfied with facility visits and glad or very glad about receiving home visits. Further adding to the complexity, respondents had differing opinions on the same cadre of providers, depending on whether the services were provided in a facility or during a home visit. This finding is in line with existing literature on the nature of power dynamics and territoriality [40–41].

An approach used by Alhassan et al. [42] found that facilitating systematic community engagement (SCE) interventions around quality of care enabled existing community groups to reflect on perceived quality of care, using 10 pre-defined domains detailed on a community scorecard. Community feedback was shared with health providers who used the information to develop action plans to address gaps in perception of quality. After six months a second SCE assessment showed improvements in perceptions of quality. This low-cost, relatively simple SCE approach, which also enabled providers to reflect on motivation, safety and risk mitigation, may add value to standards-based quality improvement interventions [43–45].

### Limitations

The study findings have several limitations. We randomly selected intervention zones, comparison zones, and household participants and matched comparison and intervention zones, in accord with the posttest only, non-equivalent control group design. However, there may be true differences between the groups that we were unable to measure given the design, which was selected because project baseline data, and data on comparison zones, were not available. Further, intervention sites were all in coastal districts, while comparison districts were adjacent and inland. As with other surveys, social desirability bias is a concern. Further, we used self-report of visits to facilities, home visits, and exposure to community events as measurement variables. Although not feasible due to financial and time constraints, as well as concerns for the quality of service statistic data, a stronger design would have included a record review of service utilization and health outcomes. The SBM-R assessments may have been prone to the Hawthorne effect, although any bias would be expected to be similar in intervention and comparison zones. The Rosenthal effect may have led to the assessors encouraging intervention zone participants more than those in comparison zones. We attempted to mitigate this by not assigning assessors to CHPS zones where they had previously worked. Further, by nature of the intervention, comparison zone participants had no prior exposure to the assessment tool. Generalizability of the findings to other locations may be limited because the project was implemented in six coastal districts of Western Ghana.

## Conclusion

While the evaluation demonstrated improved access to quality care in zones that used a standards-based approach, there were limited differences between intervention and comparison zones in clients’ experiences of care. As Ghana and other countries scale-up UHC, a pragmatic approach such as SBM-R could prove useful to engage both facility- and community-based service providers as well as community members to improve provision of care. SBM-R, as a standalone quality improvement approach, can complement other government quality improvement processes or structures. As it can also be led by facilities as a self-assessment, SBM-R can be sustainable. In addition, we must continue to grapple with how to improve and accurately measure the client’s experience of care [11,46]. Further work is needed to ensure that client experience is built into the design, implementation, monitoring, and evaluation of quality improvement interventions. Tools that are more sensitive for measuring and interpreting experience of care are needed for both monitoring and evaluation [47] so that countries like Ghana can ensure that the care clients receive meets their needs and contributes to Sustainable Development Goal 3—“ensure healthy lives and promote well-being for all at all ages” [9].

## Supporting information

S1 Table Results of bivariate and multivariate analyses of percentage of verification criteria achieved for assessment areas. (DOCX)

S1 Tool SBM-R assessment tool. (PDF)

S2 Tool Household survey. (DOCX)

## References

[1] Lawson HJO, Essuman A. Country profile on family medicine and primary health care in Ghana. Afr J Prim Health Care Fam Med. 2016;8(1):1302 10.4102/phcfm.v8i1.1302 https://www.ncbi.nlm.nih.gov/pmc/articles/PMC5125262/ 28155326 PMC5125262

[2] World Bank. Data: World Bank Country and Lending Groups—Country Classification. Available from: https://datahelpdesk.worldbank.org/knowledgebase/articles/906519-world-bank-country-and-lending-groups Cited 5 Jul 2017.

[3] World Health Organization (WHO). Maternal mortality in 1990–2015. WHO, UNICEF, UNFPA, World Bank Group, and United Nations Population Division. Maternal Mortality Estimation Inter-Agency Group; Ghana. Available from: http://www.who.int/gho/maternal_health/countries/gha.pdf Cited 5 Jul 2017.

[4] UNICEF. The State of the World's Children 2016: A fair chance for every child. New York: UNICEF; 2016. https://www.unicef.org/publications/files/UNICEF_SOWC_2016.pdf

[5] UNDP. Ghana Millennium Development Goals: 2015 Report. Available from: http://www.gh.undp.org/content/ghana/en/home/library/poverty/2015-ghana-millennium-development-goals-report.html Cited 12 Jul 2017.

[6] Quansah E, Ohene LA, Norman L, Mireku MO, Karikari TK. Social factors influencing child health in Ghana. PLOS ONE. 2016;11(1):e0145401 10.1371/journal.pone.0145401 https://www.ncbi.nlm.nih.gov/pmc/articles/PMC4706365/ 26745277 PMC4706365

[7] Zere E, Kirigia JM, Duale S, Akazili J. Inequities in maternal and child health outcomes and interventions in Ghana. BMC Public Health. 2012;12:252 10.1186/1471-2458-12-252 22463465 PMC3338377

[8] WHO. Framework for the Implementation of the Ouagadougou Declaration on Primary Health Care and Health Systems in Africa [Internet]. Brazzaville: WHO Regional Office for Africa; 2010. Available from: http://www.afro.who.int/sites/default/files/2017-06/framework-for-implementation-ouaga-9-4-10.pdf Cited 11 Jul 2017.

[9] United Nations. Sustainable Development Goal. Progress of Goal 3 in 2017. UN Website. Available from: https://sustainabledevelopment.un.org/sdg3 Cited 12 Jul 2017.

[10] Ministry of Health, Ghana. Health Sector Medium Term Development Plan 2014–2017. Accra, Ghana: Ministry of Health; 2014. http://www.moh.gov.gh/wp-content/uploads/2016/02/2014-2017-Health-sector-medium-term-dev-plan.pdf

[11] Sobel HL, Huntington D, Temmerman M. Quality at the centre of universal health coverage. Health Policy Plan. 2016;31(4):547–9. 10.1093/heapol/czv095 26420642

[12] De Man J, Mayega RW, Sarkar N, Waweru E, Leys M, Van Olmen J, et al Patient-Centered Care and People-Centered Health Systems in Sub-Saharan Africa: Why So Little of Something So Badly Needed? International Journal of People Centered Medicine. 2016;6(3):162–173. http://www.ijpcm.org/index.php/IJPCM/article/view/591

[13] Sturmberg JP, Njoroge A. People-centred health systems, a bottom-up approach: where theory meets empery. J Eval Clin Pract. 2017;23(2):467–473. 10.1111/jep.12540 27062608

[14] WHO. Standards for Improving Quality of Maternal and Newborn Care in Health Facilities Geneva: WHO; 2016 Available from: http://apps.who.int/iris/bitstream/10665/249155/1/9789241511216-eng.pdf?ua=1 Cited11 Jul 2017.

[15] Srivastava A, Avan BI, Rajbangshi P, Bhattacharyya S. Determinants of women’s satisfaction with maternal health care: a review of literature from developing countries. BMC Pregnancy Childbirth. 2015;15:97 10.1186/s12884-015-0525-0 https://bmcpregnancychildbirth.biomedcentral.com/articles/10.1186/s12884-015-0525-0 25928085 PMC4417271

[16] Tuncalp Ö, Were WM, Maclennan C, Oladapo OT, Gülmezoglu AM, Bahl R, et al Quality of care for pregnant women and newborns–the WHO vision. BJOG. 2015; 122: 1045–1049. 10.1111/1471-0528.13451 25929823 PMC5029576

[17] Ivers N, Jamtvedt G, Flottorp S, Young JM, Odgaard-Jensen J, French SD, et al Audit and feedback: effects on professional practice and healthcare outcomes. Cochrane Database Syst Rev. 2012, 6, CD000259. 10.1002/14651858.CD000259.pub3 PMC11338587 22696318

[18] Nyonator FK, Awoonor-Williams JK, Phillips JF, Jones TC, Miller RA. The Ghana community-based health planning and services initiative for scaling up service delivery innovation. Health Policy Plan. 2005;20(1):25–34. 10.1093/heapol/czi003 15689427

[19] Kyei-Nimakoh M, Carolan-Olah M, McCann TV. Millennium development Goal 5: progress and challenges in reducing maternal deaths in Ghana. BMC Pregnancy Childbirth. 2016;16:51 10.1186/s12884-016-0840-0 26960599 PMC4784346

[20] Ghana Health Services. Community-Based Health Planning and Services: National Implementation Guidelines. Accra: Ghana Health Services; 2016. Available from: http://www.ghanahealthservice.org/downloads/ServicestoClients.pdf Cited 11 Jul 2017.

[21] Centre for Health and Social Services, Ghana. National Community-based Health Planning and Services (CHPS): A policy options advisory brief. Accra: CHeSS; 2015.

[22] Necochea E, Tripathi V, Kim YM, Akram N, Hyjazi Y, da Luz Vaz M, et al Implementation of the Standards-Based Management and Recognition approach to quality improvement in maternal, newborn, and child health programs in low-resource countries. Int J Gynaecol Obstet. 2015;130: S17–S24. 10.1016/j.ijgo.2015.04.003 26115852

[23] Necochea E, Bossemeyer D, Bluestone J. Standards-Based Management and Recognition. Baltimore: Jhpiego; 2005.

[24] WHO. Service Availability and Readiness Assessment (SARA): an annual monitoring system for service delivery. Reference Manual, Version 2.2, July 2015. Available from: http://www.who.int/healthinfo/systems/sara_reference_manual/en/.

[25] Haddad S, Fournier P, Potvin L. Measuring lay people’s perceptions of the quality of primary care services in developing countries. Validation of a 20-item scale. Int J Qual Health Care. 1998;10(2);93–104. 9690882 10.1093/intqhc/10.2.93

[26] Webster TR, Mantopoulos J, Jackson E, Cole-Lewis H, Kidane L, Kebede S, et al A brief questionnaire for assessing patient healthcare experiences in low-income settings. Int J Qual Health Care. 2011;23(3):258–68. 10.1093/intqhc/mzr019 21531989

[27] Quansah E, Ohene AL, Norman L, Mireku MO, Karikari TK. Social Factors Influencing Child health In Ghana. PLOS One. 2016;11(1):e0145401 10.1371/journal.pone.0145401 26745277 PMC4706365

[28] Kyei-Nimakoh M, Carolan-Olah M, McCann TV. Millennium development Goal 5: progress and challenges in reducing maternal deaths in Ghana. BMC Pregnancy Childbirth. 2016;16:51 10.1186/s12884-016-0840-0 26960599 PMC4784346

[29] WHO. Framework on integrated, people-centered health services. Report by the WHO Secretariat, 18 December 2015, 138th session of the World Health Assembly. Available from: http://apps.who.int/gb/ebwha/pdf_files/WHA69/A69_39-en.pdf?ua=1&ua=1

[30] Ckachi Y, Kruk ME. Quality of Care: Measuring a neglected driver of improved health. Bull World Health Organ. 2017; 95:465–472. 10.2471/BLT.16.180190 28603313 PMC5463815

[31] Awoonor-Williams JK, Sory EK, Nyonator FK, Phillips JF, Wang C, Schmitt ML. Lessons learned from scaling up a community-based health program in the Upper East Region of northern Ghana. Glob Health Sci Pract. 2013;1(1):117–33. 10.9745/GHSP-D-12-00012 25276522 PMC4168550

[32] Adongo PB, Phillips JF, Aikins M, Afua Arhin D, Schmitt M, Nwameme AU, et al Does the design and implementation of proven innovations for delivery basic primary health care services in rural communities fit the urban setting: the case of Ghana’s Community-based health Planning and Services (CHPS). Health Res Policy Syst. 2014, 12:16 10.1186/1478-4505-12-16 24690310 PMC3994228

[33] Sakeah E, McCloskey L, Bernstein J, Yeboah-Antwi K, Mills S, Doctor HV. Is there any role for community involvement in the community-based health planning and services skilled delivery program in rural Ghana? BMC Health Serv Res. 2014;14:340 10.1186/1472-6963-14-340 25113017 PMC4251607

[34] Kim YM, Banda J, Kanjipite W, Sarkar S, Bazant E, Hiner C, et al Improving performance of Zambia Defence Force ART providers: evaluation of a standards-based approach. Glob Health Sci Pract. 2013;1(2):213–27. 10.9745/GHSP-D-13-00053 25276534 PMC4168581

[35] Rawlins BJ, Kim YM, Rozario AM, Bazant E, Rashidi T, Bandazi SN, et al Reproductive health services in Malawi: an evaluation of a quality improvement intervention. Midwifery. 2013;29(1):53–9. 10.1016/j.midw.2011.10.005 22079625

[36] Donabedian A. The Definition of Quality and Approaches to Its Assessment. Ann Arbor: Health Administration Press; 1980.

[37] Salam RA, Lassi ZS, Das JK, Bhutta ZA. Evidence from district level inputs to improve quality of care for maternal and newborn health: interventions and findings. Reprod Health. 2014;11(Suppl 2):3. 25208460 10.1186/1742-4755-11-S2-S3 PMC4160920

[38] Kruk ME, Vail D, Austin-Evelyn K, Atuyambe L, Greeson D, Grépin KA, et al Evaluation of a maternal health program in Uganda and Zambia find mixed results on quality of care and satisfaction. Health Affairs. 2016;35(3):510–519. 10.1377/hlthaff.2015.0902 26953307

[39] Tancred T, Schellenberg J, Marschant T. Using Mixed Methods to evaluate perceived quality of care in southern Tanzania. Int J Qual Health Care. 2016;28(2):233–239. 10.1093/intqhc/mzw002 26823050 PMC4833203

[40] Spiers JA. The Interpersonal Contexts of Negotiating Care in Home Care Nurse-Patient Interactions. Qual Health Res. 2002;12(80);1033–1057. 10.1177/104973202236579 http://journals.sagepub.com/doi/pdf/10.1177/104973202129120430 12365587

[41] Shattell M. Nurse–patient interaction: a review of the literature. J Clin Nurs. 2004;13:714–722. 10.1111/j.1365-2702.2004.00965.x http://onlinelibrary.wiley.com/doi/10.1111/j.1365-2702.2004.00965.x/full 15317511

[42] Alhassan RK, Nketiah-Amponsah E. Design and implementation of community engagement interventions towards healthcare quality improvement in Ghana: methodological approach. PLOS ONE. 2016 6(49)1–13. 10.1186/s13561-016-0128-0 PMC5081980 27785769

[43] Alhassan RK, Nketiah-Amponsah E, Spieker N, Arhinful DK, Ogink A, van Ostenberg P, Rinke de Wit TF. Effect of community engagement interventions on patient safety and risk reduction efforts in primary health facilities: evidence from Ghana. PLOS One. 2015;10(11): 1–19. 10.1371/journal.pone.0142389 PMC4664410 26619143

[44] Alhassan RK, Spieker N, Nketiah-Amponsah E, Arhinful DK, Rinke de Wit TF. Impact of community engagement interventions on frontline health workers' perspectives on Ghana's national health insurance scheme. BMC Health Services Research. 2016;16(192): 1–11. 27236330 10.1186/s12913-016-1438-y PMC4884385

[45] Alhassan RK, Spieker N, Nketiah-Amponsah E, Arhinful DK, Rinke de Wit TF. Assessing the impact of community engagement interventions on health worker motivation and experiences with clients in primary health facilities in Ghana: a randomized cluster trial. PLOS One. 2016;11(7): 1–19. 10.1371/journal.pone.0158541 PMC4954663 27439012

[46] Akachi Y, Kruk ME. Quality of Care: measuring a neglected driver of improved health. Bull World Health Organ. 2017; 95:465–472. 10.2471/BLT.16.180190 28603313 PMC5463815

[47] Haddad S, Fournier P, Potvin L. Measuring lay people’s perceptions of the quality of primary care services in developing countries. Validation of a 20-item scale. Int J Qual Health Care. 1998;10(2):93–104. 9690882 10.1093/intqhc/10.2.93

