# Supplementary material for: Can a standards-based approach improve access to and quality of primary health care? Findings from an end-of-project evaluation in Ghana
Source: PLoS One. 2019 May 10;14(5):e0216589. doi: 10.1371/journal.pone.0216589 (PMC6510430; doi:10.1371/journal.pone.0216589)
Supplement: S1 Table — (DOCX) [file pone.0216589.s001.docx]

**Supporting Table 1: Results of Bivariate and Multivariate Analyses of Percentage of Verification Criteria Achieved for Assessment Areas**

|  | **Mean % of Verification Criteria Achieved (Standard Deviation)** | | **Adjusted Analysis^a^** | | |
| --- | --- | --- | --- | --- | --- |
|  | **Intervention**  **(n=12)** | **Comparison**  **(n=12)** | **Risk Ratio** | **95% CI** | **P-Value** |
| **Facility Readiness & Management** |  |  |  |  |  |
| *Community Health Officer* |  |  |  |  |  |
| Planning, implementing and managing activities | 77.3% (25.9) | 54.5% (41.7) | 1.28 | 0.9 - 1.8 | 0.182 |
| Logistics management | 85.4% (15.4) | 74.1% (14.8) | 1.18 | 1.0 - 1.4 | 0.015* |
| Financial management | 70.5% (20.3) | 56.4% (17.9) | 1.22 | 0.9 - 1.7 | 0.19 |
| Data collection, reporting, analysis and use | 81.0% (26.2) | 47.6% (20.1) | 1.60 | 1.2 - 2.2 | 0.004** |
| *District Health Management Team* |  |  |  |  |  |
| Management of CHPS implementation^b^ | 99.2% (1.0) | 58.2% (24.4) | – | – | – |
| Support to CHO^b^ | 79.0% (6.7) | 46.3% (10.1) | – | – | – |
| *Equipment, Supplies, and Drugs* |  |  |  | - |  |
| Necessary equipment and supplies are available | 78.8% (14.1) | 73.5% (16.2) | 1.06 | 0.9 - 1.3 | 0.458 |
| **Clinical Services** |  |  |  |  |  |
| *Community Health Officer* |  |  |  |  |  |
| Child Health |  |  |  |  |  |
| Immunization | 94.6% (7.7) | 65.8% (26.2) | 1.42 | 1.2 - 1.8 | <0.001*** |
| Breastfeeding, growth monitoring, and nutrition | 95.7% (8.0) | 77.9% (17.0) | 1.22 | 1.0 - 1.5 | 0.033* |
| Acute care of infants and children | 88.2% (15.7) | 64.1% (18.1) | 1.34 | 1.0 - 1.7 | 0.026* |
| Reproductive Health |  |  |  |  |  |
| Family Planning | 88.9% (16.5) | 68.2% (23.8) | 1.29 | 0.9 - 1.8 | 0.114 |
| HIV/AIDS and other sexually transmitted infections | 97.7% (6.0) | 81.1% (17.3) | 1.18 | 1.0 - 1.4 | 0.076 |
| Antenatal care | 88.9% (12.1) | 64.9% (23.5) | 1.35 | 1.1 - 1.7 | 0.013* |
| Safe emergency delivery and newborn resuscitation | 73.95 (32.0) | 64.5% (35.0) | 1.14 | 0.8 - 1.6 | 0.462 |
| Postnatal care and essential newborn care | 83.1% (17.3) | 81.0% (20.1) | 1.02 | 0.9 - 1.2 | 0.824 |
| Illness |  |  |  |  |  |
| Infection prevention | 97.0% (7.7) | 73.5% (17.7) | 1.28 | 1.1 - 1.5 | 0.007** |
| Malaria | 98.0% (3.5) | 84.3% (14.7) | 1.14 | 1.0 - 1.3 | 0.02* |
| Tuberculosis | 88.5% (16.1) | 56.0% (30.7) | 1.53 | 1.1 - 2.1 | 0.012* |
| Hypertension | 99.3% (2.6) | 85.6% (20.3) | 1.14 | 1.1 - 1.2 | <0.001*** |
| Diabetes | 100.0% (0) | 65.2% (27.6) | 1.45 | 1.2 - 1.8 | 0.002** |
| Sickle cell disease | 100.0% (0) | 72.2% (23.5) | 1.36 | 1.2 - 1.5 | <0.001*** |
| Elephantiasis/lymphatic filariasis | 91.0% (15.3) | 50.0% (37.1) | 1.72 | 1.1 - 2.7 | 0.022* |
| Mental health | 94.0% (15.7) | 69.7% (36.4) | 1.36 | 1.0 - 1.9 | 0.083 |
| Diarrhea | 98.8% (4.4) | 86.5% (18.9) | 1.13 | 1.0 - 1.2 | 0.005** |
| Intestinal worms | 97.3% (6.9) | 71.3% (29.8) | 1.32 | 1.1 - 1.6 | 0.009** |
| Acute respiratory tract infections | 92.5% (16.8) | 81.1% (29.1) | 1.09 | 0.9 - 1.3 | 0.379 |
| First aid and home emergencies | 89.2% (14.6) | 70.8% (13.7) | 1.25 | 1.1 - 1.4 | <0.001*** |
| ^a^ Multivariate model controlled for facility maturity, staff-to-population ratio, and clustering at the district level.  ^b^ Criteria asked of DHMTs use non-parametric model due to small sample size.  * p-value < 0.05, ** p-value < 0.01, ***p-value < 0.001 | | | | | |
