# Supplementary material for: Can a standards-based approach improve access to and quality of primary health care? Findings from an end-of-project evaluation in Ghana
Source: PLoS One. 2019 May 10;14(5):e0216589. doi: 10.1371/journal.pone.0216589 (PMC6510430; doi:10.1371/journal.pone.0216589)
Supplement: S1 Tool — (PDF) [file pone.0216589.s002.pdf]

Empowering Communities
for Better Health

STAR CHPS ASSESSMENT

Community Health Management Committee tool

Funded by:

Implemented by:

STAR CHPS mission is to strengthen communities and boost superior performance and efficiency at CHPS Zones in the 6 coastal districts in the Western Region, thus increasing access to high quality primary healthcare to over a million residents.

District:

CHPS Zone:

Assessor(s): ***Date:***

Compendium of Performance Standards for Community Health Planning and Services (CHPS) *(Draft updated 11/29/2013)*			
**Categories and Thematic Areas**	**Total standards**	**Total standards achieved**	**%**
**1. Standards for Community Health Officer**			
**A. Basic Clinical Services**			
**A1. Child Health**			
1. Immunization	5		
2. Nutrition, Breastfeeding and Growth Monitoring	8		
3. IMNCI – Children less than 2 months up to 5 years	9		
**A2. Reproductive Health**			
4. Family Planning	5		
5. HIV and STI	9		
6. Antenatal Care	10		
7. Safe Emergency Delivery and Newborn Resuscitation	6		
8. Post-natal Care and Essential Newborn Care	4		
**A3. Illnesses and Emergencies**			
9. Infection Prevention	7		
10. Malaria	4		
11. TB	2		
12. Hypertension	2		
13. Diabetes	2		
14. Sickle cell	2		
15. Elephantiases	2		
16. Mental Health	2		
17. Diarrhoea	2		
18. Intestinal Worms	2		
19. Acute Respiratory Tract Infections	2		
20. First Aid and Home Emergencies	11		
**1. Community Linkages and Outreach Services**			
21. Health Promotion and Health Education	5		

22. Disease Surveillance	4		
23. Home Visits	2		
24. School Health	6		
25. Outreach Activities	2		
26. Supporting Community Health Volunteers	1		
27. Working with the Community Health Committee	4		
**2. Management of Activities, Services and Logistics**			
28. Planning, implementing and managing activities	2		
29. Logistics Management	5		
30. Financial Management	8		
31. Data collection, reporting, analysis and use	5		
**32. Standards for Community Health Volunteers**			
32. Disease prevention and environmental sanitation	1		
33. Home visiting	4		
34. Home management of minor ailments	2		
35. Community Outreaches	7		
36. Equipment, logistics and supplies	2		
**37. Standards for Community Health Committee**			
37. Governance, membership and operation	5		
38. Selection and supervision of community volunteers	2		
39. Welfare of CHO (include Security)	2		
40. Facility maintenance	2		
41. Resource mobilization and management	3		
**42. Standards for District Health Management Team (DHMT)**			
41. Management of CHPS Implementation	9		
42. Support to CHO	3		
43. Equipment	3		

Gaps

Performance standard Number	Gaps identified

ACTION PLAN

Gap Identified	Action to be taken	Person(s) responsible	Support needed	When will action be completed

District Health Management Team: 42) CHPS Implementation 43) CHO Support 44) CHPS Implementation Steps

42	Management of CHPS Implementation		Yes, No, N/A	Comments
42.1	Develops annual workplan for CHPS	**Verify by observation / interview:**		
		A written plan for CHPS implementation		
		Schedules support visits to CHPS Zones		
		Mobilizes and allocates resources to CHPS Zones		
		Organizes performance reviews		
42.2	Has institutionalized key actions to support the achievement of the CHPS milestones	**Verify by observation / interview / record review how the DHMT has engaged actively to implement the following actions:**		
		Planning for CHPS implementation		
		Community entry process		
		Community health compound		
		Provision of Essential Equipment		
		Posting of CHOs		
		Volunteers deployment		
42.3	Plans and conducts supervision of CHPS Zones	**Verify by observation / interview:**		
		Develops bi-annual schedule for supervision		
		Informs CHPS Zone of impending visit		
		Prepares all logistics needed to undertake the visit (checklist, SOPs, mobilization of logistics, etc.)		
		Conducts supervision by engaging in in-depth supervision and on-the-job training using appropriate tool		
		Documents all findings		
		Writes report		
42.4	Takes actions in the CHPS implementation process	**Verify by observation / interview / record review:**		
		Knows the exact status (achievement of steps) of each CHPS		
		Organizes quarterly meetings with CHOs, CHMCs and CHVs		
		Develops bi-annual schedule for supervision		
		Outlines achievements and challenges		

		Develops action plans to solve challenges		
		Writes minutes of meetings		
		Sets date for next meeting		
**42.5**	**Outlines the progress on the 15 implementation steps for each CHPS zone**	**Verify by interview / observation that CHMC:**		
		Plans for CHPS have been developed		
		Communities have been consulted and health workers sensitized		
		Health workers have had (a series of) dialogue sessions with community leadership		
		Community information durbars have been held		
		CHOs have been selected and trained		
		Community Health Committee members have been selected and oriented a durbar has been held for their approval		
		A Community Profile has been compiled		
		Community Health Compound has been constructed		
		Logistics for placing the CHO in the community have been mobilized		
		A Durbar has been held to formally launch the CHO program		
		Community Health Volunteers have been selected		
		Durbar have been held for the approval of CHV		
		The CHVs have been trained		
		Logistics (Resources) have been mobilized for equipping of Community Health Volunteers		
		A Durbar has been organized for approval of Community Health Volunteers		
**42.6**	**Community Health Management Committee members are selected according to protocol**	**Verify by observation / interview / record review that the following are members of the CHMC:**		
		CHO		
		Representative from traditional council		
		A herbalist		
		Teacher		
		TBA		
		Volunteer (optional)		
		Chemical Seller		
		Local agricultural association leader (Fishermen or farmers' association, etc.)		
		Community environmental health and sanitation representative (or its equivalent)		
		Assembly member		
		Women's group representative		
		Opinion leader		

		Muslim or Religious representative		
42.7	**CHVs are selected according to protocol**	**Verify by interview / observation:**		
		CHMC members hold consultations with Community Chief to identify community youth with good character from good homes to be trained as volunteers		
		CHMC members conduct background search on probable candidates and make suggestions for the consideration of the Chief		
		CHMC members consult with the Chief to vet the names		
		Candidates are shortlisted for presentation at a durbar for approval		
		CHMC members organize a community durbar to present the shortlisted candidates for approval		
		CHMC members liaise with health authorities to ensure the approved volunteers are trained		
		CHMC members continue to liaise with the CHO to ensure training of the Volunteers and their presentation at post-training durbar where their roles, responsibilities, dos and don’ts are spelled out to everyone		
42.8	**DHMT ensures that CHPS Zones are accredited to NHIS**	**Verify by interviewing/observing**		
		DHMT applies to NHIS for accreditation of CHPS zone		
		DHMT pays for the fee to NHIS		
		DHMT ensures that all requirements needed for the accreditation is put in place		
		DHMT invites NHIS team to inspect the facility		
		Facility is accredited if they meet the the required criteria		
42.9	**DHMT assists CHPS Zones with logistics**	**Verify by interviewing/observing**		
		DHMT/Sub-districts estimates logistics for CHPS zones on the following: Equipment; Drugs; Supplies; Vaccines		
		CHPS zones informed to request for the items		
		CHO sends requisition to DHMT to countersigning and authorization		
		CHO receivers logistics from store		
		CHO documents items received in the appropriate books and tallies		
43	**Support to CHO**			
43.1	**Organizes orientation workshops and study tour for the newly posted CHN to become CHOs**	**Verify by observation / interview:**		
		Train all the CHNs before they are deployed into the CHPS zones		
		Lists of trainings held available		
		Lists of participants who took part in the training		
		Report on the training held		
43.2	**CHOs in the zones are provided with comfort items**	**Verify by observation / interview:**		
		Room furniture: living room set, dining room set		
		TV		
		Radio		
		Kitchen Ware: Sets of plates and cups; Sets of cooking ware; Set of cutlery		
		Refrigerator (gas or electricity)		

43.3	**CHOs in the zones are provided with logistics and equipment**	**Verify by observation / interview:**		
		Working gear (Boot and Rain coat)		
		Stethoscope		
		Consumables		
		Thermometer		
		Angiopoid Lamp		
		Weighing Scales: Bathroom and Salter Hanging Scale		
		Training Manuals & Dissemination		
		IE& C materials		
		Cold boxes		
		Transport: motorbike, bicycle, boat		
		Boat logistics (life jackets, torch, kerosene stove, mattress, camp bed, mega phone)		

DHMT: Achievement of 15 Steps

	**15 Steps**	**Name of CHPS:** yes / no	Details / comments	**Name of CHPS:** yes / no	Details / comments	**Name of CHPS:** yes / no	Details / comments
1	Plans for CHPS have been developed						
2	Communities have been consulted and health workers sensitized						
3	Health workers have had (a series of) dialogue sessions with community leadership						
4	Community information durbars have been held						
5	CHOs have been selected and trained						
6	Community Health Committee members have been selected and oriented a durbar has been held for their approval						
7	A Community Profile has been compiled						
8	Community Health Compound has been constructed						
9	Logistics for placing the CHO in the community have been mobilized						
10	A Durbar has been held to formally launch the CHO program						
11	Community Health Volunteers have been selected						
12	Durbar have been held for the approval of CHV						
13	The CHVs have been trained						
14	Logistics (Resources) have been mobilized for equipping of Community Health Volunteers						
15	A Durbar has been organized for approval of Community Health Volunteers						

Empowering Communities
for Better Health

STAR CHPS ASSESSMENT

District Health Management Team Tool

Funded by:

Implemented by:

STAR CHPS mission is to strengthen communities and boost superior performance and efficiency at CHPS Zones in the 6 coastal districts in the Western Region, thus increasing access to high quality primary healthcare to over a million residents.

Compendium of Performance Standards for Community Health Planning and Services (CHPS)<br>*(Draft updated 11/29/2013)*			
**Categories and Thematic Areas**	**Total standards**	**Total standards achieved**	**%**
**1. Standards for Community Health Officer**			
**A. Basic Clinical Services**			
**A1. Child Health**			
1. Immunization	5		
2. Nutrition, Breastfeeding and Growth Monitoring	8		
3. IMNCI – Children less than 2 months up to 5 years	9		
**A2. Reproductive Health**			
4. Family Planning	5		
5. HIV and STI	9		
6. Antenatal Care	10		
7. Safe Emergency Delivery and Newborn Resuscitation	6		
8. Post-natal Care and Essential Newborn Care	4		
**A3. Illnesses and Emergencies**			
9. Infection Prevention	7		
10. Malaria	4		
11. TB	2		
12. Hypertension	2		
13. Diabetes	2		
14. Sickle cell	2		
15. Elephantiases	2		
16. Mental Health	2		
17. Diarrhoea	2		
18. Intestinal Worms	2		
19. Acute Respiratory Tract Infections	2		
20. First Aid and Home Emergencies	11		
**1. Community Linkages and Outreach Services**			
21. Health Promotion and Health Education	5		

22. Disease Surveillance	4		
23. Home Visits	2		
24. School Health	6		
25. Outreach Activities	2		
26. Supporting Community Health Volunteers	1		
27. Working with the Community Health Committee	4		
**2. Management of Activities, Services and Logistics**			
28. Planning, implementing and managing activities	2		
29. Logistics Management	5		
30. Financial Management	8		
31. Data collection, reporting, analysis and use	5		
**32. Standards for Community Health Volunteers**			
32. Disease prevention and environmental sanitation	1		
33. Home visiting	4		
34. Home management of minor ailments	2		
35. Community Outreaches	7		
36. Equipment, logistics and supplies	2		
**37. Standards for Community Health Committee**			
37. Governance, membership and operation	5		
38. Selection and supervision of community volunteers	2		
39. Welfare of CHO (include Security)	2		
40. Facility maintenance	2		
41. Resource mobilization and management	3		
**42. Standards for District Health Management Team (DHMT)**			
41. Management of CHPS Implementation	9		
42. Support to CHO	3		
43. Equipment	3		

Gaps

Performance standard Number	Gaps identified

ACTION PLAN

Gap Identified	Action to be taken	Person(s) responsible	Support needed	When will action be completed

Community Health Management Committee: 37) Governance 38) CHVs 39) CHO Welfare 40) Facility Care 41) Resource Mobilization

37	Governance, membership and operation		Yes, No, N/A	Comments
37.1	CHMC members recognize the importance of CHPS to the community	**Verify by interview / observation:**		
		Provides appropriate information to community members to enable them adopt healthy lifestyle.		
		Facilitate transportation of those who are sick and need further services		
37.2	Member(s) know and are able to state their responsibilities	**Verify by interview / observation:**		
		supervise health volunteers		
		liaise between the community and the health sector		
		ensure welfare of CHVs and the CHO		
		speak for and ensure better health conditions within the community		
		resolve conflict in the community when conflicts arise between the community members and those working on CHPS		
		mobilize the community members to obtain and maintain CHPS compound		
		ensure security of the CHO		
		mobilize resources to support the CHPS program at the community level		
		co-ordinates waste management at the CHPS compound		
		support health programs such as health education, disease surveillance and advocacy at the community level		
		manage local finances at the level of the volunteers establishment		
		facilitate arrangements for Emergency Transport Response Arrangements at the community level		
		members meet regularly		
37.3	Minutes of meeting are recorded accurately with decisions and follow up actions carefully noted	**Verify by interview / observation:**		
		Minutes of the CHMC meetings are Dated, Timed and signed		
		Attendance or participants list of the CHMC meetings is available		
		CHMC meeting minutes document the Purpose and Agenda of the meeting		
		CHMC meetings have developed Action Plans with clearly assigned responsibilities and timelines		
		CHMC minutes indicate the date of the next meeting		
37.4	Health programs and activities are supported by committee	**Verify by interview / observation:**		
		Supports the CHO in effecting referrals by facilitating Emergency Response Transport Arrangements.		
		Provides support to the CHO by identifying and designating a helper to clean the community health compound		
		Encourages CHVs to trace defaulters		
		Visits CHO during outreach activities (optional)		

		The committee members play the role of community key informants in disease surveillance		

37.5	Conflicts are resolved successfully	**Verify by interview / observation:**		
		CHMC members listens carefully to both sides of the issue and do not take rash decisions		
		CHMC members make appointments to meet each side of disputing party to hear each side of the case objectively		
		CHMC members are impartial in all cases		
		CHMC members document decisions and roles expected of them and all contesting parties		
**38**	**Selection and supervision of community volunteers**			
38.1	**Administrative supervision of work of community volunteers is undertaken appropriately**	**Verify by interview / observation:**		
		CHMC members coordinate and administer the operations of the Volunteers in support of the CHO (Keeps a work plan / Supervision schedules)		
		Logistics and supply issues of the Volunteer are handled by the CHMC members		
		Disputes, behavioral aberrations and conflicts with the Volunteers engagement are documented and resolved by the CHMC members		
		CHMC members document performance (administrative) of the CHVs		
		Keeps a work plan for supervision and monitoring of the community health volunteers		
38.2	**The committee provides appropriate motivation for volunteers**	**Verify by interview / observation:**		
		Ensures that CHV is provided with working tools (Rain coat, Torch Light, Back-Pack, Bicycles, Condoms, Paracetamol, Foaming tablets etc.)		
		CHMC members ensure that due recognition is given to hard working CHVs at durbars or festivals		
		Visits volunteers regularly		
**39**	**Welfare of CHO (include Security)**			
39.1	**The committee cares for the welfare of the CHO**	**Verify by interview / observation:**		
		ensure that the CHO is comfortable (They facilitate the procurement of comfort items such as Mattress, radio and /or Television set)		
		CHMC members organize communal labor to plant wood lots for shade and to serve as wind breaks around the Community Health Compound		
		Facilitate the connection of the Community Health Compound to the National Electricity Grid or Solar Panel lighting		
		Liaises with the Districts Assembly and other institutions to support the work of the CHO		
		CHMC members organize communal labor to plant wood lots for shade and to serve as wind breaks around the Community Health Compound		
39.2	**The security of the CHPS compound is managed appropriately**	**Verify by interview / observation:**		
		All doors and windows at the CHPS are in good condition and under lock and key		
		Provides 24-hour or night security at the CHPS		
**40**	**Facility & service maintenance**			
40.1	**Waste created at the CHPS compound is managed appropriately**	**Verify by interview / observation:**		
		Outside bins are available at the CHPS compound and are covered		
		Safety boxes are available and being used		

		Waste disposal structures in place at the CHPS (Ask and check how waste is disposed at compound)		
40.2	**A Community ambulance system / emergency transportation system is created and maintained**	**Verify by interview / observation:**		
		Type of transport system in place		
		Minutes of meetings held with Transport owners to discuss transport arrangements are available		
		List of emergency transport owners with names and contact numbers exist		
		pre-agreed payment methods for emmergency transport (in writing, preferred)		
**41**	**Resource mobilization and management**			
41.1	**Records of contributors and contributions to the CHPS compound are kept**	**Verify by interview / observation:**		
		An updated logbook/register exists that documents all donations to support the community health compound		
		Register on contributors/donations captures the following:		
		personal details of person making or reciving payment: name, age, sex, address		
		What the person has contributed / received (if payment made)		
41.2	**Accurate records are kept of contributions and payments made to support the CHPS compound**	**Verify by interview / observation:**		
		Date of contribution		
		Sex of contributor		
		Age of contributor		
		Description of what was contributed		
		Signature of contributor		
41.3	**All financial transactions are recorded appropriately**	**Verify by interview / observation:**		
		Date of transaction		
		Description of transaction		
		Money in		
		Money out		
		Balance		
		Signature of person doing the transaction		

STAR CHPS

Empowering Communities
for Better Health

STAR CHPS ASSESSMENT

Community Health Volunteer Tool

Funded by:

Implemented by:

STAR CHPS mission is to strengthen communities and boost superior performance and efficiency at CHPS Zones in the 6 coastal districts in the Western Region, thus increasing access to high quality primary healthcare to over a million residents.

District:_______________________________

Compendium of Performance Standards for Community Health Planning and Services (CHPS) *(Draft updated 11/29/2013)*			
**Categories and Thematic Areas**	**Total standards**	**Total standards achieved**	**%**
**1. Standards for Community Health Officer**			
**A. Basic Clinical Services**			
**A1. Child Health**			
1. Immunization	5		
2. Nutrition, Breastfeeding and Growth Monitoring	8		
3. IMNCI – Children less than 2 months up to 5 years	9		
**A2. Reproductive Health**			
4. Family Planning	5		
5. HIV and STI	9		
6. Antenatal Care	10		
7. Safe Emergency Delivery and Newborn Resuscitation	6		
8. Post-natal Care and Essential Newborn Care	4		
**A3. Illnesses and Emergencies**			
9. Infection Prevention	7		
10. Malaria	4		
11. TB	2		
12. Hypertension	2		
13. Diabetes	2		
14. Sickle cell	2		
15. Elephantiases	2		
16. Mental Health	2		
17. Diarrhoea	2		
18. Intestinal Worms	2		
19. Acute Respiratory Tract Infections	2		
20. First Aid and Home Emergencies	11		
**1. Community Linkages and Outreach Services**			
21. Health Promotion and Health Education	5		

22. Disease Surveillance	4		
23. Home Visits	2		
24. School Health	6		
25. Outreach Activities	2		
26. Supporting Community Health Volunteers	1		
27. Working with the Community Health Committee	4		
**2. Management of Activities, Services and Logistics**			
28. Planning, implementing and managing activities	2		
29. Logistics Management	5		
30. Financial Management	8		
31. Data collection, reporting, analysis and use	5		
**32. Standards for Community Health Volunteers**			
32. Disease prevention and environmental sanitation	1		
33. Home visiting	4		
34. Home management of minor ailments	2		
35. Community Outreaches	7		
36. Equipment, logistics and supplies	2		
**37. Standards for Community Health Committee**			
37. Governance, membership and operation	5		
38. Selection and supervision of community volunteers	2		
39. Welfare of CHO (include Security)	2		
40. Facility maintenance	2		
41. Resource mobilization and management	3		
**42. Standards for District Health Management Team (DHMT)**			
41. Management of CHPS Implementation	9		
42. Support to CHO	3		
43. Equipment	3		

Gaps

Performance standard Number	Gaps identified

ACTION PLAN

Gap Identified	Action to be taken	Person(s) responsible	Support needed	When will action be completed

Community Health Volunteer: 32) Disease prevention and Environmental Sanitation 33) Home Visiting 34) Home Management 35) Community Outreach 36) Logistics

32	**Disease Prevention and Environmental Sanitation**		**Yes, No, N/A**	**Comments**
**32.1**	**CHV conducts community observation (i.e. walk around) and situation analysis with CHO and CHMC**	**Verify by observation / interview:**		
		Makes plan for a community walk around		
		Informs community leaders about the purpose of the intended walk about		
		Captures findings from field visits in field note book		
		Sketches Community map to indicate areas that need health action		
		Engages with community people (one-on-one discussion/ group discussions/in durbars) to find out their views on health concerns in the community		
		Maps out community water source, refuse dump, markets, kind of toilet facilities, drainage system, environmental risk areas for mosquitos to breed		
		Disseminates findings		
		Name of CHV in minutes from planning meeting		
**33**	**Home visiting**			
**33.1**	**Preparation is made for conducting home visits**	**Verify by observation / interview:**		
		CHV plans with CHO for visits to be conducted during the month		
		CHV prepares home visiting bag		
**33.2**	**Home is entered appropriately**	**Verify by observation / interview:**		
		Declares his or her presence by knocking or a locally appropriate greeting to signal his presence		
		CHV waits for response		
		Enters home and greets household members		
		Asks for head of household or family		
		Establishes rapport, introduces himself and purpose of visit		
		Seeks permission to carry out purpose		
**33.3**	**Conducts a household needs assessment**	**Verify by observation / interview:**		
		Identifies household members and verifies and notes individual status and wellbeing (presence / absence / well-being; for women in child-bearing age, pregnancy status/lactating etc.)		
		Identify family members with special needs e.g. mentally handicapped, acutely ill		
		Checks for house hold membership and updates in community register if necessary		

		Asks if anyone in the home sleeps under ITNs		
		Refers cases beyond his/her ability to CHO or Health Facility according to protocol (Examine his referral booklet)		
**33.4**	**Ends visit by exiting home appropriately**	**Verify by observation / interview:**		
		Reminds members of households/family on issues discussed and other issues of concern to the family		
		Asks clients to summarize what has been discussed to ascertain their understanding		
		asks for any outstanding concerns and respond appropriately		
		Plans next visit with hosts		
		Thanks host/family for their time		
**34**	**Home management of minor ailments**			
**34.1**	**Children with fever are identified and managed appropriately within the home**	**Verify by observation / interview:**		
		Assesses if child has fever/is hot by placing the back of his or her hand over the child's forehead (if the body temperature of child is higher than that of the CHV/if the child feels hot to touch, there is an indication that the child has fever)		
		reduces fever by tepid sponging AND / OR a wet towl under armpits and on forehead Soaks a towel in water and gently wipes child from the feet towards head		
		Gives child paracetamol syrup after sponging as follows: 0-6months give 2.5 mls; 6months-1year give 5mls; 1 year-2 years give 7.5mls; 2years to 5years give 10mls; 6year to 12 year give 1 tablet (500mg); 12 years and above give adult dose – (1000mg)		
		Ensures client has eaten (fluid and sugar)		
		ACT is given according to weight in divided doses (morning & evening) for three consecutive days		
		gives first dose to children in his/her presence		
**34.2**	**Diarrhea is identified and managed**	**Verify by observation / interview:**		

	**correctly**	Asks client or care giver when diarrhea started and how often stools are passed		
		Ask client if stool contains blood		
		Determines the level of dehydration in the child: feeling and testing for the elasticity of the skin, level of sunkeness of eye socket, level of dryness of the tongue		
		teach mother / caregiver how to mix and administer ORS (note: expiry date, clean water, correct amount of water for sachet), cautioning that a new solution should be mixed after 24 hour period		
		show mother how to administer Zinc and continue for 10 days (infants: dissolve tablet in small amount of breastmilk; children: disolve in small amount of clean water or can be chewed) Zinc tablet dosage: 10 mg for 6mths-11mths and 20mg for 12mths-5yrs		
		advises client to give ORS after child has loose stools or vomits		
		Counsels breastfed child to be breastfed frequently		
		Refer if necessary		
35	**Community Outreach**			
35.1	**CHV participates in outreach activities**	**Verify by observation / interview:**		
		Informs community of date and venue of planned outreach visit		
		Announces the presence of the CHO/outreach team in the community		
		Assists with preparation of the venue		
		CHV attends outreach activities arranged by CHO		
		assists CHOs e.g. health education, weighing children, recording data		
35.2	**The community is mobilized for health education**	**Verify by observation / interview:**		
		plans activities, suitable date and time for program with CHMC		
		Identifies target groups/ social groups and topics to be discussed with them		
35.3	**Facilitates health education session in the community**	**Verify by observation / interview:**		
		Welcomes community people to gathering		
		introduces the program and / or presenter		
		facilitates discussion and questions		
35.4	**Health education is provided to promote breastfeeding**	**Verify by observation / interview:**		
		Breast milk is the natural food for the baby		
		Breast milk is clean because it is not contaminated by dirty hands or any other materials.		
		Breast milk protects the baby from infection		

		Breast milk is always available and requires no preparation		
		Provides information that breast milk provides all the necessary nutrients for the baby to grow and develop well		
		Provides information that breast milk also establishes a special relationship between mother and the baby		
		exclusive breastfeeding can be used as a family planning method for the first 6 months (LAM)		
**35.5**	**Information is provided on the various family planning methods that are available (individual or group)**	**Verify by observation / interview:**		
		**Barrier methods:** Condoms for male and females, Spermicides or foaming tablets,		
		**Short term (Short-acting) methods:** oral contraceptive pills (combined and mini pill), injectable (Depo Provera, Norigynon-3monthly), Lactation Amenorrhea Method (LAM) and Natural FP methods.		
		**Long term (Long –Acting reverisble) methods:** either intra uterine device or implants; Jadelle		
		**Permanent / irreversible methods:** which include Tubal Ligation(female sterilization) or Vasectomy (male sterilization)		
**35.6**	**Benefits of family planning are discussed with an individual, couple or group**	**Verify by observation / interview:**		
		Provides information that FP enables the mother to remain healthy and strong		
		Provides information that FP helps children grow healthy and strong		
		Provides information that FP enables parents to feed and take good care of their children.		
		Provides information that FP helps prevent STIs (condoms)		
		Provides information that the use of FP helps avoid unwanted pregnancies		
**35.7**	**Demonstration and education on condoms using a model and condom is provided for an individual, couple or group**	**Verify by observation / interview:**		
		Ensures that condoms are not expired and not damaged and are safe to use		
		Tears open		
		Holds the tip of the condom squeeze out the air		
		Places the condom on the dummy penis and to rolls down		
		Leaves space at the tip of the condom to collect semen during ejaculation		
		Advises that penis is fully withdrawn out of vagina soon after sex		
		Removes and ties condom up, wrap for disposal		

		Advises on proper disposal of used condoms		
**36**	**Logistics**			
**36.1**	**Equipment**	**Verify by inspection:**		
		Community register		
		Back Pack (bag)		
		Wellington boots		
		Identity t-shirts		
		Malaria flipchart		
		health education materials (i.e. Posters)		
		Condom model		
		Raincoat		
		Thermometer (digital)		
		Torch light with batteries		
**36.2**	**Home visiting bag supplies**	**Verify by inspection CHV has adequate quantities of the following for at least one month:**		
		ORS		
		Zinc tablets - at least 1 dose / 14 tablets		
		Adults ACTs (AA, AL) - at least 1 dose		
		Paediatric ACTs (AA, AL) - at least 1 dose		
		Paracetamol Tablets and Syrup/suppository		
		Condoms		
		Alcohol rub		
		Cotton bandage		
		Cotton wool swabs		
		Crepe bandage		
		Disposable gloves		
		Gauze swabs		
		Gentian violet lotion		
		Hand Sanitizer		
		Hand towels		
		Insecticide Treated Nets (ITNs)		
		Liquid soap		
		Measuring cup		
		Methylated spirit		
		Pen		
		Pencil		
		Plaster		
		Plastic apron		
		Plastic file		

		Plastic galipot		
		Plastic Sheet		
		Scissors		
		Small note book		
		Spoon		

Empowering Communities
for Better Health

STAR CHPS ASSESSMENT

Community Health Officer Tool1

Funded by:

Implemented by:

STAR CHPS mission is to strengthen communities and boost superior performance and efficiency at CHPS Zones in the 6 coastal districts in the Western Region, thus increasing access to high quality primary healthcare to over a million residents.

District:________________________________

Compendium of Performance Standards for Community Health Planning and Services (CHPS) *(Draft updated 11/29/2013)*			
**Categories and Thematic Areas**	**Total standards**	**Total standards achieved**	**%**
**1. Standards for Community Health Officer**			
**A. Basic Clinical Services**			
**A1. Child Health**			
1. Immunization	5		
2. Nutrition, Breastfeeding and Growth Monitoring	8		
3. IMNCI – Children less than 2 months up to 5 years	9		
**A2. Reproductive Health**			
4. Family Planning	5		
5. HIV and STI	9		
6. Antenatal Care	10		
7. Safe Emergency Delivery and Newborn Resuscitation	6		
8. Post-natal Care and Essential Newborn Care	4		
**A3. Illnesses and Emergencies**			
9. Infection Prevention	7		
10. Malaria	4		
11. TB	2		
12. Hypertension	2		
13. Diabetes	2		
14. Sickle cell	2		
15. Elephantiases	2		
16. Mental Health	2		
17. Diarrhoea	2		
18. Intestinal Worms	2		
19. Acute Respiratory Tract Infections	2		
20. First Aid and Home Emergencies	11		
**1. Community Linkages and Outreach Services**			
21. Health Promotion and Health Education	5		

22. Disease Surveillance	4		
23. Home Visits	2		
24. School Health	6		
25. Outreach Activities	2		
26. Supporting Community Health Volunteers	1		
27. Working with the Community Health Committee	4		
**2. Management of Activities, Services and Logistics**			
28. Planning, implementing and managing activities	2		
29. Logistics Management	5		
30. Financial Management	8		
31. Data collection, reporting, analysis and use	5		
**32. Standards for Community Health Volunteers**			
32. Disease prevention and environmental sanitation	1		
33. Home visiting	4		
34. Home management of minor ailments	2		
35. Community Outreaches	7		
36. Equipment, logistics and supplies	2		
**37. Standards for Community Health Committee**			
37. Governance, membership and operation	5		
38. Selection and supervision of community volunteers	2		
39. Welfare of CHO (include Security)	2		
40. Facility maintenance	2		
41. Resource mobilization and management	3		
**42. Standards for District Health Management Team (DHMT)**			
41. Management of CHPS Implementation	9		
42. Support to CHO	3		
43. Equipment	3		

Gaps

Performance standard Number	Gaps identified

ACTION PLAN

Gap Identified	Action to be taken	Person(s) responsible	Support needed	When will action be completed

Child Health: 1) Immunization, 2) Nutrition and 3) IMNCI

1	Immunization		Yes, No	Comments
1.1	**Mothers / Caretakers bringing children for immunizations receive accurate information and education**	**Verify by interviewing/observing**		
		Checks immunization record and determines which vaccination should be given		
		Provides reasons for the vaccination		
		Emphasizes the importance of having ALL the appropriate cycles of vaccinations		
		Clarifies when to come in for the next immunization sessions		
		Discusses possible side effects and how to manage (e.g. pain, fever, etc.)		
		Answers any questions and checks understanding / comprehension		
1.2	**Monthly coverage of immunization is displayed graphically in the CHPS**	**Verify by interviewing/observing**		
		Has prepared the monthly coverage of immunization in a graphical form		
		Has displayed the graphs on the board in the CHPS compound.		
		Graphs displayed include information from at least 12 months previous		
1.3	**Vaccines are stored properly**	**Verify by interviewing/observing the following:**		
		Vaccines are properly stored inside the refrigerator		
		The refrigerator temperature sheet is available and kept up to date with recording twice a day		
		Vaccines are maintained with expiry date, batch numbers, stock levels		
		Vaccine ledger is maintained with stock level		
1.4	**The provider properly prepares for vaccine administration:**	**Verify by interviewing / observing:**		
		Washes hands with soap and water and dries them, or uses alcohol hand rub		
		Explains to the mother or caretaker how to hold the child		
		Explains to the mother or caretaker which vaccination the child will receive		
		Checks for correct vaccine		
		Checks for right dose		
		Checks the expiration date on the vaccine		
1.5	**Immunizations are administered correctly**	**Verify by interviewing / observing:**		
		Correctly administers oral vaccination to the baby/child, ensuring the baby has swallowed all		
		For injectable vaccination: correctly draws up correct solution from correct vial		
		Wipes the injection site with clean water (if needed)		

		Pinches up the anterior thigh (babies) or deltoid muscle (children) to inject, and inserts the needle/syringe		
		Injects the medication and withdraws the needle quickly		
		Discards the needle and syringe together in appropriate container (never recaps the needle)		
		Washes hands with soap and water and dries them, or uses alcohol hand rub		
		Records the information on the appropriate forms and the vaccination card		
		Thanks the mother and the child		
**2**	**Breastfeeding, Growth Monitoring, and Nutrition**			
**2.1**	**CHO/midwife educates communities (community, group, couple) or the individual client on infant feeding**	**Verify that the CHO / midwife is able to :**		
		**Give four advantages** of exclusive breast feeding for the infants first six months of life: 1. gives natural immunity; 2. is easily and efficiently digested; 3. prevents pregnancy by delaying menstrual cycle (LAM); 4. safe and costs less; 5. helps bonding between baby and mother; 6. prevents breast infection; 7. promotes development of baby		
		**Give three disadvantages** of formula / substitution feeding: 1. increases incidence of diarrhea; 2. increases chance of Respiratory Tract Infections due to low immunity; 3. is expensive; 4. may cause milk allergy/intolerance; 5. interferes with bonding; 6. pre-disposes the mother to early return of menstrual cycle/fertility		
		Counsels the mother on how to correctly position the baby for breastfeeding, encouraging the mother sit upright in a comfortable position while keeping the baby's head and body in a straight line (supporting the spine)		
		Ensures the baby is facing the breast with the baby's nose is opposite her nipple		
		Mother is to hold the breast with her hand – four fingers under the breast and the thumb positioned on top of the breast near the areola and use this to guide the nipple towards the baby		
		Supports the mother to attach the baby to the breast - touching the baby's lips with her nipple, waiting until the baby opens the mouth wide and then moving the baby quickly to the breast		
		Ensure the baby is properly fixed with the areola visible above the baby's upper lip, lower lip turned outwards, and baby's chin touching the breast		
**2.2**	**Education is provided on how to express breast milk**	**Verify that the CHO/midwife educates the mother on the steps for expressing breast milk:**		
		Wash hands thoroughly		
		Sit or stand comfortably and hold a clean container with a fitted lid underneath the breast		
		Put first finger and thumb on either side of the areola, behind the nipple		

		Press slightly inward toward the breast between the finger and thumb, expressing milk, until the milk flow becomes slow		
		Repeat the same with the other breast, alternating breasts for 20 to 30 minutes		
		Stored expressed milk in the container with a well fitting lid or cover		
**2.3**	**CHO / Midwife identifies and manages conditions of the breast which may interfere with breastfeeding**	**Verify that the CHO / Midwife is able to:**		
		Correctly **identify four common condition**s which may interfere with breastfeeding: inverted nipples, sore and cracked nipples, breast engorgement, mastitis (inflammation of breast), and breast abscess		
		Correctly counsels women with inverted nipples: to ensure the baby attaches to areola not the nipple & advise on using a syringe to assist with getting nipples to protrude before feeding		
		Correctly counsels women with sore and cracked nipples to expose nipple to air, continue breast feeding on the least affected breast, ensure correct attachment, and/or apply Gentian Violet paint, 0.5 % if candidiasis		
		Correctly counsels women with breast engorgement to apply warm compresses, to continue breast feeding , and to extract milk		
		Correctly counsels women with mastitis to apply warm compresses, provide medication for pain relief and refer		
		Indicates that women with breast abscess should be referred		
		**Provides three examples** of how the mother can prevent common breast conditions: give 1st breast milk soon after delivery, use various positions for breast feeding, attach baby correctly to breast, breastfeed on demand, empty one breast at a time, and do not use artificial nipples		
**2.4**	**Health education is provided on the importance of growth monitoring**	**Verify that the CHO / midwife demonstrates knowledge regarding why growth monitoring is essential:**		
		It helps in knowing the nutritional status and the general health of the child		
		It helps in early detection of growth failures and early correction		
		It is a forum to educate mother on the importance of good feeding, immunization and other issues related to the health of a child		
**2.5**	**Children and babies are weighed correctly**	**Verify by observing / interviewing that the CHO / midwife correctly weighs the child:**		
		Hang up the scale securely at a height and the face of the scale at the eye level of the nurse		
		Check scale to see if the arrow is on zero (with weighing pant if applicable)		

		Ask mother to remove all clothing on the infant or child		
		If weighing a child assist the mother to put the child in the weighing pant, put the loop of the pant over the hook of the scale, and make sure the child's feet are hanging without touching anything **OR** if weighing an infant, place the baby in the scale with legs stretched		
		Read the weight of the child on the scale when the child is calm		
		Write down the weight in kilograms with figures to the hundred gram (e.g. 5.225 kg)		
**2.6**	**CHO correctly records on the growth monitoring card and the relevant trend is noted**	**Verify by interviewing / observing:**		
		Records month and year of visit		
		Beginning with the month of birth write out the following month of the year in the following box		
		Record the weight of the child by plotting dot on the line corresponding to the weight in kilogram		
		Join the dot to the previous month to obtain the growth curve		
		Upward direction curve = Good; child growing well		
		Horizontal direction = Alert; not gaining weight		
		Downward direction = Alarm; losing weight – take immediate action		
**2.7**	**CHO / midwife identifies malnourished children**	**Verify that the CHO / midwife is able to :**		
		**Provide at least two signs / symptoms** of Marasmus in children: looks like an "old man", is always hungry, has little muscle or fat and is very thin, and bone in face/chest stand out		
		**Provide at least two signs/ symptoms** of Kwashiorkor in children; 1. arms looks very thin and wasted; 2. face looks very puffy due to oedema; 3. bilateral pitting oedema		
		**Provide at least two signs/ symptoms** of Marasmic Kwashiorkor in children: 1. hair silky /thin and looks reddish; 2. skin depigmentation and dermatitis especially on the legs; 3. the child is not hungry but miserable and looks dejected		
**2.8**	**CHO / midwife provides health education on prevention and control of malnutrition in children**	**Verify that the CHO / midwife provides correct information:**		
		Give a variety of foods, enriching porridge with pounded fish, groundnut and beans		
		Mash food to make it soft, do not add hot spices (e.g. pepper) to the child's food, and give fruits with meals		
		Feed frequently (at least 2-4 times depending on the age of the child)		
		Increase amount of food as child grows and supervise feeding if child is old enough to eat on his/her own		
		Food and utensils used for cooking and serving must be clean		

		Encourage regular growth monitoring in children		
**3**	**Acute care of infants and children**			
3.1	**Good interpersonal communication skills are maintained throughout the consultation**	**Verify by interviewing / observing the following:**		
		Greets the caretaker or parent		
		Encourages the parent/caretaker to ask questions		
		Uses language the caretaker (and child) understands		
		Checks the mother / caretaker's understanding		
**3.2**	**Initial information on child is obtained from the caretaker / mother**	**Verify by interviewing / observing:**		
		Name		
		Age (in months)		
		Sex		
		Presence of fever, cough, diarrhea, or ear problem		
		Assesses breastfeeding status		
		Checks the immunization status		
		Asks what are the child's problems		
		Asks if it is initial or follow-up visit		
**3.3**	**Initial assessment/physical examination of sick child is conducted**	**Verify by observing / interviewing whether the CHO aks and checks the following by using IMNCI chart booklet**		
		Check for very severe disease and need for immediate referral (general danger signs)		
		Washes hands with soap and water and dries them, or uses alcohol hand rub		
		Weighs the child, noting low weight for age		
		Checks temperature		
		Checks pulse		
		Checks respiratory rate (breaths per minute)		
		Checks for jaundice in the eyes, skin, palms soles		
		Checks for pallor and other signs of anemia		
		Check the child's immunization status		
		Records the findings in the child's clinical record / card		
**3.4**	**Correctly identifies, classifies and manages the infant's/child's illness: jaundice**	**Verify by interviewing / observing:**		
		identifies severe jaundice = jaundice in under-24-hour child or jaundice in palms/soles		
		treats severe jaundice by urgent referral and treatment to prevent low blood sugar		
		identifies jaundice = after 24 hours of age and palms/soles are **not** yellow		
		treats jaundice = Counsels mother to give home care and return immediately if palms/soles become yellow		

		Records the classification in the child's clinical record		
3.5	**Correctly identifies, classifies and manages the infant's/child's illness: <u>acute diarrhea</u>**	**Verify by interviewing / observing:**		
		checks for sunken eyes		
		assesses hydration by doing skin pinch		
		identifies severe dehydratioin = presence of two signs (movement only when stimulated/no movement, sunken eyes, very slow skin pinch return)		
		manages severe dehydration = plan C if no comorbidities OR refer urgently to hospital if additional 'severe' classification		
		identifies mild dehydration = presence of two signs (restless, irritable, sunken eyes, slow skin pinch return)		
		manages mild dehydration = plan B and counsel mother to return immediately if not improving after 2 days OR refer urgently if another 'severe' classification		
		identifies no dehydration = if client does not have two conditions indicative of dehydration		
		manages no dehydration = plan A (fluid, zinc, supplements and fluid) at home and counsels to return in 5 days if not improving		
		Records the classification in the child's clinical record		
3.6	**Correctly identifies, classifies and manages the infant's/child's illness: <u>acute respiratory illness</u>**	**Verify by interviewing / observing:**		
		assesses if child is breathing too fast = >50 breaths per minute if 2-12 months, >40 in 1-5 year olds		
		assesses if child has chest indrawing, stridor, or wheezing		
		identifies severe pneumonia/very severe disease = history of coughing/breathing difficulty and any warning sign OR chest indrawing OR stridor in a calm child		
		identifies pneumonia = fast breathing		
		manages severe pneumonia / very severe disease or pneumonia = give first dose of antibiotics AND /OR refers		
		identifies cough / cold = no signs of pneumonia or very severe disease		
		manages wheezing child = refer		
		manages child with cough > 3 weeks or recurrent wheezing = referral for assessment for TB or asthma		
		Records the classification in the child's clinical record		
3.7	**Correctly identifies, classifies and manages the infant's/child's illness: <u>fever</u>**	**Verify by interviewing / observing:**		
		assesses for stiff neck, runny nose, swollen throat		
		assesses for signs of measles over the last three months = rash, red eyes, coughing		
		identifies very severe febrile disease = child with warning sign or stiff neck		

		manages very severe febrile disease = give first dose of quinine for malaria AND give first dose of an appropriate antibiotic AND treat the child to prevent low blood sugar AND give one dose of paracetamol for high fever (>38.5C) AND urgently refer		
		identifies malaria = fever without runny nose, no signs of meales, and no other visible cause of fever		
		manages malaria = give appropiate dose of ACTs AND paracetamol if high fever (>38.5C) AND review follow-up with the caregiver (return in 2 days if fever persists)		
		identifies fever - malaria unlikely = runny nose or other visible cause of fever with no signs of measles		
		manages fever - malaria unlikely = give one dose of paracetamol if high fever (>38.5C) and review follow-up with caregiver (return in 2 days if fever persists)		
		Records the classification in the child's clinical record		
**3.8**	**Correctly identifies, classifies and manages the infant's/child's illness: <u>measles</u>**	**Verify by interviewing / observing:**		
		identifies severe complicated measles= signs of measles and any warning sign or clouding of cornea/extensive mouth ulcers		
		manages severe complicated measles = give vitamin A treatment AND give first dose of appropriate antibiotic AND refer urgently		
		identifies measles with eye or mouth complications = signs of measles AND pus draining from the eye OR mouth ulcers		
		manages measles with eye or mouth complications = give vitamin A treatment, if applicable treat eye infection (tetracycline) or mouth ulcer (gentian violet) AND follow-up in 2 days		
		identifies measles = signs of measles without other complication		
		manages measles = give vitamin A		
		Records the classification in the child's clinical record		
**3.9**	**Correctly identifies, classifies and manages the infant's/child's illness: <u>ear pain / discharge</u>**	**Verify by interviewing / observing:**		
		Assesses swelling or pus draining in/behind ear		
		identifies mastoiditis = tender swelling behind the ear		
		manages mastoiditis = give first dose of appropriate antibiotic AND give first dose of paracetamol AND urgently refer		
		identifies acute ear infection = pus draining from ear less than 14 days		
		manages acute ear infection = give appropriate antibiotic for 5 days AND give paracetamol AND follow-up in 5 days		
		identifies chronic ear infection = pus draining from ear more than 14 days		
		manages chronic ear infection = refer		

Reproductive Health: 4) Family Planning; 5) HIV/AIDS and STIs; 6) Antenatal Care; 7) Safe Emergency Delivery and Newborn Resuscitation; 8) Post-natal Care and Essential Newborn Care

4	**Family Planning**		**Yes, No, N/A**	**Comments**
4.1	**The CHO/midwife counsels clients appropriately on family planning choices**	**Determined by interviewing/observing if the CHO/midwife:**		
		Ensures there is adequate space and privacy (auditory and visual) to counsel clients on family planning		
		Shows respect to the client and encourages her to explain needs, concerns, and ask any questions during the session		
		Counsels the client about importance of spacing births: the best thing to do for her health is to space birth to pregnancy (24 months) and birth to birth (36 months)		
		Discusses **all** family planning options available, including hormonal contraceptives (COC, injectables, implants), condoms, IUD, lactational amenorrhea method (LAM), mini-laparotomy		
4.2	**The CHO provides the client information about hormonal family planning methods (COC and injectable)**	**Verify by observing/interviewing that the CHO discusses private with the client:**		
		Brands available and how often different methods are taken: COCs daily at the same time, Norigynon monthly, Noristerat every 2 months, and Depo-Provera every 3 months		
		How the hormonal methods prevent pregnancy and effectiveness of each method		
		**At least one advantage and** disadvantage of COCs: fertility returns almost immediately after stopping the pill, pill decreases menstrual flow and cramps, pill protects against ovarian and endometrial cancer; **disadvantage** - requires daily use, does not protect against STIs including HIV		
		**At least one advantage and** disadvantage of injectables: decrease menstrual flow and cramps, protects against ovarian and endometrial cancer, easy to use; **disadvantage** - delay in return to fertility, may cause abnormal bleeding, does not protect against STIs including HIV		
		**At least three common** side effects of COCs: nausea, dizziness, breast tenderness, headaches, weight change, bleeding between periods		
		**At least three common** side effects of injectables: irregular bleeding/spotting between periods or amenorrhea, weight gain, nausea, dizziness, breast tenderness, headaches, mood changes		

		**All warning signals** for clients on any hormonal contraceptive**:** abdominal pain, chest pain, blurred vision, severe headache or leg pain, heavy bleeding		
4.3	**The CHO / midwife ensures there are no contra-indications to initiating clients on hormonal family planning methods**	**Verify that the CHO / midwife determines if the client is medically eligble to take combined oral contraceptives (COC) or injectables:**		
		Not currently pregnant: <7 days since last menstrual cycle **OR** abstinence since last menses **OR** correctly and consistently using a contraceptive method **OR** is within 4 weeks postpartum **OR** if < 6 months postpartum and is fully breastfeeding and no onset of menses **OR** referral for pregnancy test		
		No undiagnosed vaginal bleeding		
		Not breastfeeding a baby less than 6 weeks (injectable) **OR** 6 months old (COC)		
		Not less than 21 days postpartum in non-breastfeeding women chosing COC		
		No active liver disease (hepatitis or tumors), severe headaches/migrains, or breast cancer		
		No diabetes of > 20 years duration, history of heart disease or stroke, or uncontrcolled high blood pressure (>140/90mm Hg)		
		Drug interactions: rifampicin or anticonvulsants		
4.4	**Clients are started on their preferred method and given appropriate education/information on follow-up**	**Determine by if the CHO/midwife properly educates the client by interviewing / observing:**		
		Using samples, describes how and when to start COC or injectable: in first 1-7 days of menstrual period; if after day 7, use backup method or abstain for one week		
		Describes how to continue with COC: start a new pack immediately after finishing the previous pack; if forgot to take a pill, take immediately when remember then take the next pill at the usual time. This means you may take two pills in one day. If a pill is not taken on two or more days in a row, continue with the pack if there are 7 or more active pills. If there are less than 7 active pills, discard and start a new pack immediately. Always use a back up method when a pill is missed.		
		Explains the need to use condoms or abstain from sex 7 days in case of severe diarrhea or vomiting for at least 24 hours while taking COC		
		Describes how to continue with injectables: return to the clinic for the injection every 3 months for DMPA, every 2 months for NET-EN or every month if using the monthly injection		
		Explains what is necessary if the client is late for the next injection		
		At end of the session, checks client's understanding, schedules the next visit indicating that she should return if she misses her period for 2 months while on COC or any time she has questions or concerns		
		Documents in the client's record and the facility register		
4.5	**The CHO administers injectable contraceptives appropriately**	**Observe in the procedure area if the CHO:**		
		Prepares supplies: new sterile needle, syringe; unexpired vial of DMPA, NET-EN, or Noriygnon; **and** cotton swabs		
		Explains the procedure to the client		
		Correctly draws up the injectable contraceptive		

		Adheres to infection prevention practices: undertakes hand hygiene and wipes the injection site with clean water		
		Gives the injection appropriately: inserts the needle deep into the deltoid or gluteus muscle, pulls back the plunger to ensure no blood visible, injects drug, **and** removes needle - applying pressure to injection site with cotton		

		Disposes needle and syringe in a puncture-resistant container, without removing, recapping or breaking the needle		
		Hand hygiene is undertaken after injection is given		
**5**	**HIV/AIDS and STIs**			
**5.1**	**Community, group and individual education is provided on how HIV is transmitted**	**Verify by interviewing / observing if the following information is discussed:**		
		sexual contact - oral, vaginal, and anal intercourse with an infected person		
		contaminated blood and body fluids - sharing sharps and needles contaminated with blood during shaving, barbering, scarification, tattooing, tribal marks, etc		
		contaminated blood - transfusion of infected blood		
		Pregnant woman with HIV can transmit the virus to her child during pregnancy, childbirth, or through breast milk		
**5.2**	**Demonstrates and provides education on condom use**	**Verify by observing / interviewing that the CHO educates the client on key information on condom use:**		
		Check condom carefully for expiry date and any damage before removing it from the wrapper		
		Open the packet from the rough edge, careful not to tear the condom while opening		
		Place the condom on the dummy penis and roll down, leaving space at the tip for collection of semen during ejaculation		
		Remove the penis, with the condom on, from the vagina before it becomes soft by holding the condom firmly at the base so as not to spill the semen		
		Properly dipose of the condom by tying it and either burning or throwing away in a latrine		
**5.3**	**Prepares client for testing, providing key information on HIV testing and counseling, emphasizing the benefits of knowing ones status**	**Verify by interview / observation that the provider:**		
		Greets client and assures confidentiality		
		Describes the benefits and risks of HIV testing		
		Describes why to get tested		
		Describes when to get tested?		
		Describes how the test works and what the testing process is		
		Provides information on where the closest place to get tested is		
**5.4**	**Prepare to perform rapid HIV test**	**Verify by interview / observation:**		
		Briefly explains the procedure to the client to let them know what to expect		
		Prepares the supplies, marking test with client information per site policy		
		Washes hands with soap and water and dries hands		
		Puts on a pair of clean examination latex gloves		
**5.5**	**HIV rapid test is performed**	**Verify by interview / observation:**		
		Collects peripheral blood as per standards for test		
		Draws small amount (one – two drops) of blood serum onto the test as required		
		Applies one – two drops of buffer solution to test strip / kit / cartridge		
		Waits for correct amount of time (as per the test specifications) for test to develop		

		Interprets the results according to the national test algorithm = if test non-reactive, reports negative test result OR if test is reactive, performs second test using another rapid test, if second test is reactive, reports positive test result, if result of second test is non-reaactive, reports as indeterminate and refers for lab testing		

		Needles and syringes are disposed in a sharps container		
		Washes hands with soap and water and dries them.		
		Records test results		
5.6	**The test results are given to the client**	**Verify by interview / observation that the provider:**		
		Tells the client that the test result is positive / negative		
		Explains the meaning of positive / negative test results		
		Assists client to express their concerns about positive / negative test results		
		Explores client's intended actions in relation to concerns		
		Helps client identify with whom they can share their test results		
		Encourages health promoting behaviors including good nutrition, sleep and avoidance of drugs and alcohol		
		Stress the need for follow-up appointments and seeing prompt health care when sick		
5.7	**Health education is provided to community, couples and individuals on STIs and how to prevent them**	**Verify by observation / interview that the provider:**		
		Describes **at least two common symptoms** of STIs, which increase risk for HIV: uretheral/vaginal discharge, genital ulcers, inguinal swelling, scrotal swelling, lower abdominal pain		
		Describes **at least two common adverse effects** of STIs: neonatal eye infections, infertility, ectopic pregnancies, still birth		
		Educates on how to prevent STIs - by staying with one sexual partner		
		Educates on how to prevent STIs - use condoms during sex		
		Encourages client to seek early health care when experiencing the signs and symptoms		
		Describes ways to reduce risk factors		
5.8	**Conducts an appropriate physical assessment in clients with signs and symptoms of STIs**	**Verify by interview / observation that the provider:**		
		Washes hands with soap and water and dries them, or uses alcohol hand rub		
		Puts on examination gloves		
		examines the male client appropriately: inspects the perineum, scrotum, penis/urethra, AND lymph nodes in the inguinal region		
		examines the female client appropriately: inspects the vulva and vaginal opening for discharge		
		Records findings		
5.9	**Clients with STIs are managed appropriately**	**Verify by interview / observation that the provider:**		
		Prescribes appropriate treatment for clients presenting symptoms OR refers client appropriately		
		Counsels the client		
		Completes required documentation (referral form and/or register)		
		Discuss follow up with client to ensure treatment compliance		
6	**Antenatal Care**			
6.1	**Health history is obtained during**	**Verify by observation / interview that the provider:**		

	**client consultation**	Greets the pregnant woman and her husband/companion cordially/respectfully AND ensures privacy during the visit		
		An initial rapid evaluation of the pregnant woman is conducted at the first contact - assess for danger signs		
		Reviews obstetrical history		
		Reviews medical history		
		Determines need for tetanus toxoid immunization		
		Asks for current use of tobacco, alcohol or other harmful substances		
		Reviews ABO and RH factor - need for testing or previous results		
		Determines the expected date of delivery and gestational age		
		Documents findings in the maternal health record book		
**6.2**	**Anemia is identified and managed**	**Verify by observation / interview that the provider:**		
		Identifies **at least three symptoms of anemia:** fatigue, loss of energy, rapid heart beat, shortness of breath, dizziness, history of abundant bleeding during menstrual periods		
		Identifies **at least two signs of anemia:** conjunctival pallor, pale palms, weak pulse, elevated heart rate, elevated respirator rate (>20 per minute)		
		Conducts diagnostic test for anemia - hemoglobin test OR refers		
		Manages the client according to Hemoglobin test results: Hb 7-11g/dL = investigates possible causes of anemia and addresses them according to anemia protocol OR referral if Hb <7 g/dL		
		Documents findings in the maternal health record AND/OR referral document		
**6.3**	**Hypertension is identified and managed**	**Verify by observation / interview that the provider:**		
		Identifies **at least two symptoms of uncontrolled hypertension: s**evere headache, blurred vision, dizziness, upper abdominal pain		
		Measures blood pressure		
		Manages the client according to BP: systolic or diastolic blood pressure above 130 / 90 mmHg, respectively, refers or reassures client if below these parameters		
		Performs simple proteinuria test (Use multistix)		
		Manges client with proteinuria: refer		
		Explains the findings to client and subsequent follow-up		
		Documents findings in the maternal health record AND/OR referral document		
**6.4**	**Fetal disorders are identified and managed**	**Verify by observation / interview that the provider:**		
		Performs abdominal inspection to check for fetal movement and measures fundal height		
		Conducts appropriate physical exam according to gestational age: listens to fetal heart rate if ≥ 20 weeks AND/OR determine fetal lie and presentation by palpation if ≥ 36 weeks		
		Manages the client with abnormal findings appropriately: referral if no fetal movements OR fundal height below expected OR fetal heart rate below/over expected, ORmalpresentation		

		Documents findings in the maternal health record AND/OR referral document		
**6.5**	**PMTCT activities are implemented for women living with HIV** *(conduct Standards 5.1-5.6 during ANC visit for HIV)*	**Verify by observation / interview that the provider:**		
		Provides necessary support to the pregnant woman and encourage partner referral for HIV testing		
		Educates the woman on use of ARVs for prevention of transmission of HIV to her child AND ART for her own health if she needs it		
		Assesses the HIV positive pregnant woman - obtains specimen to conduct CD4 test OR refers to appropriate clinic offering HIV services		
		Provides ARV or ART according to the woman's needs OR refers to appropriate clinic		
		Counsels the woman on infant feeding: exclusive BF until 6 months, complimentary feeds from 6 months, BF until 12 months OR exclusive formula for 6 months, promote good hygiene and use of clean water		
		Documents findings in the maternal health record AND/OR referral document		
**6.6**	**Tuberculosis is identified and mana**	**Verify by observation / interview that the provider:**		
		Identifies **at least four signs/symptoms of TB**: history of coughing for more than one week, weight loss, night sweats, fever, chest pain, or contact with a person diagnosed with TB		
		Manages the symptomatic client appropriately: if symptomatic take sputum sample according to guidelines OR refer		
		Indicates the importance of follow-ing up the result of the sputum smear, irrespective of where it is obtained		
		Documents findings in the maternal health record AND/OR referral document		
**6.7**	**Identifies, prevents, and manages malaria in pregnancy**	**Verify by observation / interview that the provider:**		
		Provides **at least three signs/symptoms of malaria in pregnancy**: asymptomatic, fever, headache, shivering,		
		Counsels on use of LLIN every night AND **at least two other measures** to take to reduce risk of malaria transmission: wear protective clothing, use repellants, manage the environment, avoid being outside late at night		
		If malaria is suspected, performs Rapid Diagnostic Test		
		If RDT is negative and malaris still suspected, request microscopy laboratory diagnosis		
		If malaria not suspected, educates the woman on the importance of taking IPT during pregnancy		
		Provides appropriate antimalarial - Malaria Intermittent Preventive Treatment (SP) for women without malaria OR appropriate treatment for women with malaria according to national guidelines OR referral if severe malaria		
		Recommends to return for IPTp as needed, and if being treated for malaria, emphasizes the need to finish taking all medicines , as prescribed		
		Documents findings in the maternal health record AND/OR referral document		

6.8	**Identifies, prevents, and manages syphilis in pregnancy**	**Verify by observation / interview that the provider:**		
		Provides **at least two signs/symptoms of syphilis**: sore (chancre), headache, pains, rash, lymph node swelling, patchy hair loss		
		Screens/ tests the woman for syphilis with Rapid Diagnostic Test OR other test/referral if not available		
		Educates the woman on syphilis as an STI and consequences of untreated syphilis in pregnancy		
		Manages the woman appropriately - treats according to national guidelines if screen positive OR referral OR reassurance		
		Documents findings in the maternal health record AND/OR referral document		
6.9	**Individualized general care and counseling based on findings and protocols is provided to the pregnant woman**	**Verify by observation / interview that the provider:**		
		The client's safety is ensured by good infection prevention practices during consultation and care		
		Gives Ferrous Folate (60 mg Iron and 400 mcg Folic acid) once daily giving enough to last until next visit;		
		Counsels about eating food rich in Vitamin C, and avoiding tea, coffee and colas		
		Confirms tetanus vaccination status and gives tetanus toxoid (TT) if due		
		Treats for helminths (Worms): gives mebendazole, 500mg OR Albendazole		
		Explains findings from the clinical history, physical examination and available laboratory tests using easy-to-understand language		
		Provides specific advice and counseling to the woman and her husband or companion as needed (i.e., common discomfort, rest, safe sex, nutrition, hygiene and breast feeding)		
		Develops / reviews birth plan and Complication readiness plan with the woman, including preparations for normal birth and eventual emergencies		
		Documents findings in the maternal health record AND/OR referral document		
6.10	**Counsels client on pregnangy management (Counsels Pregnant woman on abnormal conditions)**	**Verify by interview / observe that the provider:**		
		Counsels client on managing abdominal pain AND constipation and hemorrhoids in pregnancy - positioning on side with pillow between knees, massage painful area AND increase intake of fresh fruits, vegetables, and fluids and void sitting for long periods		
		Counsels client on managing swollen ankles and feet: elevate legs and feet when sitting or lying, avoid crossing legs or standing for long periods		
		Counsels client on **at least three ways to** manage nausea / vomiting: 1. eat plain dry Biscuits/Crackers; 2. eat small frequent meals; 3. avoiding overeating; 4. drink ginger or lemon in warm water to reduce the nausea; 5. sit up after meals; 6. avoid odours that induces the nausea; 7. ; 8. brush teeth / chew chewing stick after eating		
		Counsel client that despite common local beliefs, meat, eggs, snail and okra **should not be avoided** during pregnancy		
		Counsel client that despite common local beliefs attending ANC clinic early does not cause still birth		

		Explains to the client the benefits of healthy timing and spacing of pregnancy		
		Provides information on **at least three post-partum contraceptive choices:** lactational amenorrhea, progestin-only injectables/pills, IUCD, tubal ligation, and cobmined oral contraceptives if not breastfeeding		
		Counsels the client about the proper use of other family planning methods during the postpartum, including immediate postpartum methods such as the IUCD or tubal ligation		
7	**Safe Emergency Delivery and Newborn Resuscitation**			
7.1	**Immediate assessment of the laboring pregnant woman is conducted at first contact**	**Determine by interview / observation if the provider:**		
		Asks for woman's antenatal card or other information if available about the history of this pregnancy and other pregnancies		
		Identifies/inquires with the woman about **at least four danger signs: 1. v**aginal bleeding; 2. difficulty breathing; 3. fever,; 4. severe headaches; 5. blurred vision; 6. convulsions/fits; 7. fainting		
		Inquires if and when membranes "bag of waters" ruptured , including color		
		Washes hands with soap and water and air dry or uses alcohol hand rub before conducting physical exam		
		Takes the woman's vital signs: BP, Pulse, Resp, AND Temp		
		Times the intensity and duration of 2 contractions together		
		Determines the lie AND descent of foetal head		
		Listens to the foetal heart for one full minute. If suspects foetal distress (foetal heart rate is $<$ 110 beats per minute or $>$ 150 beats per minute), turns mother on her left side		
		Records all information on partograph AND/OR maternal health record		
7.2	**The laboring woman is prepared for delivery**	**Determine by interview / observation that the provider:**		
		Prepares basic supplies and equipment: delivery set AND neonatal resuscitation equipment/SKAMGOA AND dry towels/blankets AND oxytocin		
		Washes hands , wears gloves and puts on protctive clothing		
		Explains to woman that she is fully dilated and that she is almost ready to start pushing		
		Assists woman to assume the birthing position of her choice after ensuring her bladder is empty		
		Swabs vulva and prepares area for delivery		
		Puts a clean mackintosh under her buttock and another cloth on her abdomen		
		Monitors for signs of second stage		
7.3	**The second stage of labor is carefully monitored and supported (Midwife)**	**Determine by interview / observation that the midwife:**		
		Visually monitors the decent of the head AND the foetal heart rate after each contraction		
		Observes duration and interval of contractions during a 10 minute period		
		Takes and documents vital signs: BP and pulse every 15 mins and respirations every 30 mins		
		Urges mother to push with each contraction, but only when she has the urge to		
		Offers fluids at least every 2 hours		

		Gives constant emotional support		
		Records all information on partograph		
7.4	**The baby is delivered**	**Determine by interview / observation that the midwife:**		
		Assists the woman to push in a controlled manner, asking the mother not to push after the delivery of the head		
		Avoids harmful practices during delivery: avoids massage of the vaginal walls, does not give enema, avoids fundal pressure, avoids episiotomy unless clinically indicated		
		Feels for the cord around the neck, if slightly tight then slip over the shoulders		
		Allows for rotation of the head, then delivers anterior shoulder, posterior shoulder, and the rest of the body, noting the time of delivery		
		Delivers baby onto mother's abdomen		
		Gently wipes, dries and wraps baby		
		Immediately asseses the newborn: APGAR within first minute AND determine if baby needs resuscitation		
		Clamps and cuts the cord within 3 minutes OR when the cord stops pulsating		
		APGAR score of the newborn is determined 5 minutes after delivery		
7.5	**A newborn who is not breathing after delivery is resuscitated**	**Determine by observing demonstration that the provider knows how to resuscitate infant:**		
		Clears airway, mouth first and nose if necessary		
		Positions the baby with neck slightly extended and on a firm surface, keeping baby dry and warm		
		Selects appropriate mask AND properly positions on baby's face over chin, mouth and nose, forming a seal		
		Pumps wh ile checking seal: squeeze bag 2 to 3 times		
		Observes for the rise of the chest AND takes corrective action until the chest rises appropriately		
		Ventilates the new born with 30- 60 breaths per minute, stopping after every minute to look for spontaneous breathing		
		Continues ventilation until spontaneous cry/breathing begins AND baby's respiratory rate is maintained at 30-60 breaths per minute OR baby does not respond in 20 minutes		
		Ties cord and keeps baby dry and warm and continue to observe baby while attending to the mother		
		Explains condition of baby to the mother or relative		
7.6a	**(a) Active Management of the Third Stage of Labour is performed**	**Determine by observing / interviewing that the midwife:**		
		Gives 10 units of oxytocin intra muscularly in the mother's thigh within the first minute after delivery		
		Waits 2 - 3 minutes and cut the cord		

		Changes gloves		
		Feels for uterine contraction, if present, positions hand at the symphysis pubis and pushes uterus gently upwards		
		Waits for contraction and performs controlled cord traction to deliver placenta		
		Ensures uterus is contracted and massages if necessary		
		Examines the placenta and membranes for completeness		
		Swabs vulva and examines genital tract for tears		
		Cleans mother and makes her comfortable		
		Records all information on partograph		
**7.6b**	**(b) Passive management of the third stage of labor (NON-MIDWIFE)**	**Determine by observing / interviewing that the non-midwife:**		
		Gives mother oxytocin according to protocol		
		Waits 2 - 3 minutes and cuts the cord		
		Encourages mother to start breastfeeding		
		Empties the bladder		
		Carefully delivers placenta without pulling on the cord (observing signs of separation of the placenta - elongation of the cord, hardening of the uterus, trickle of blood)		
		Ensures uterus is contracted and massages if necessary		
		Examines placenta and membranes for completeness		
		Cleans mother and makes her comfortable		
		Documents all proceedings		
**8**	**Post-natal Care and Essential Newborn Care**			
**8.1**	**Immediate post natal care is provided to the mother for six hours after delivery**	**Verify by interview / observation that the provider:**		
		Infection prevention practices maintained during postnatal care: handwashing, glove use		
		Continuously massages the woman's uterus to expel clots every 15 minutes for one hour		
		Addresses the mother's questions and concerns		
		Gives the mother something to drink		
		Takes history AND conducts general examination to identify danger signs: infection, hypertensive complications, dizziness, fits, excessive bleeding, perineal swelling or tears		
		Teaches the mother and the support person the danger signs of baby and the mother: bleeding from the cord or vagina, breathing difficulty in baby, blue lips and hands of baby		
		Records temperature, pulse, blood pressure, lochia and urinary output over the six hour time period		
**8.2**	**Immediate post natal care is provided to the baby for six hours after delivery**	**Verify by observing / interviewing that the provider:**		
		Keeps baby warm and dry, covering the baby's head and feet, avoiding bath for first 6 hours		

	after delivery	Observes the baby's overall appearance: posture, muscle tone, alertness, skin color, and movements		
		Keeps baby with mother (bonding) and initiates breast feeding if not done already, starting Kangaroo Mother Care if newborn is underweight		
		Examines head, neck, eyes, face and mouth: symmetry fontanels, moulding of head, sutures, swellings, deformities, palate, lips, eye discharge		
		Examines chest: rate of breathing, retraction of chest, abnormal pulsations and any secretions from the breasts		
		Examines abdomen and cord: distension, umbilical hernia, cord for bleeding, tenderness, excretion of urine and meconium/feces		
		Examines external genitalia: deformities or indeterminate sex, undescended testis, labial adhesions, imperforate anus		
		Examines extremities: deformities (e.g. extra digits, webbed fingers or toes, joint movements, swellings over bones, club feet) or inability to move a limb		
**8.3**	**The woman and her family are provided education on the postpartum care of the mother-baby dyad**	**Determined by observing / interviewing that the provider:**		
		Educates on balanced nutrition. intake of water/other fluids, and adequate rest		
		Educates on importance of daily bath to keep genital areas and breasts clean and dry		
		Encourages emotional support from family members		
		Informs woman of family planning services available		
		Educates on importance of exclusive breast feeding		
		Educates on basic care of the newborn: keeping baby warm (head covered), allowing to sleep between feeds, bathing every 2-3 days, care of circumcised penis (males), avoid moulding of head with hot water		
		Educates on cord care: keep clean and exposed, only applying methylated spirit, wash hands before touching the cord		
		Educates on danger signs and when to immediately seek care: jaundice, cord infection (redness, purulent discharge), difficulty in feeding and fever		
		Educates on importance of immunization and growth monitoring, scheduling next clinic visit		
**8.4**	**Assessment of mother and baby is conducted on follow up postnatal visits (6 hours to 6 weeks) after delivery**	**Verify by observing / interviewing that the provider:**		
		Asks about the mother and baby's general wellbeing and if she has any questions or concerns		
		Enquires about infant feeding: ease of latching on, frequency and duration of feeds, whether the baby wakes up to feed		
		Enquires about frequency of urination, educating the mother that it should be at least 6 times in 24 hours		
		Enquires about frequency and consistency of baby’s stool, educating that it should be soft, yellow and seedy but should not be watery		
		Enquires about content and frequency of meals and fluid intake of the mother		
		Ensures the mother has taken her Vitamin A capsules		

		Is she getting enough rest?		
		Appropriately addresses any problems of the mother and child, counseling and refering as appropriate		

STAR CHPS

Empowering Communities
for Better Health

STAR CHPS ASSESSMENT

Community Health Officer Tool 2

Funded by:

Implemented by:

STAR CHPS mission is to strengthen communities and boost superior performance and efficiency at CHPS Zones in the 6 coastal districts in the Western Region, thus increasing access to high quality primary healthcare to over a million residents.

District:________________________________

Compendium of Performance Standards for Community Health Planning and Services (CHPS)<br>*(Draft updated 11/29/2013)*			
**Categories and Thematic Areas**	**Total standards**	**Total standards achieved**	**%**
**1. Standards for Community Health Officer**			
**A. Basic Clinical Services**			
**A1. Child Health**			
1. Immunization	5		
2. Nutrition, Breastfeeding and Growth Monitoring	8		
3. IMNCI – Children less than 2 months up to 5 years	9		
**A2. Reproductive Health**			
4. Family Planning	5		
5. HIV and STI	9		
6. Antenatal Care	10		
7. Safe Emergency Delivery and Newborn Resuscitation	6		
8. Post-natal Care and Essential Newborn Care	4		
**A3. Illnesses and Emergencies**			
9. Infection Prevention	7		
10. Malaria	4		
11. TB	2		
12. Hypertension	2		
13. Diabetes	2		
14. Sickle cell	2		
15. Elephantiases	2		
16. Mental Health	2		
17. Diarrhoea	2		
18. Intestinal Worms	2		
19. Acute Respiratory Tract Infections	2		
20. First Aid and Home Emergencies	11		
**1. Community Linkages and Outreach Services**			
21. Health Promotion and Health Education	5		

22. Disease Surveillance	4		
23. Home Visits	2		
24. School Health	6		
25. Outreach Activities	2		
26. Supporting Community Health Volunteers	1		
27. Working with the Community Health Committee	4		
**2. Management of Activities, Services and Logistics**			
28. Planning, implementing and managing activities	2		
29. Logistics Management	5		
30. Financial Management	8		
31. Data collection, reporting, analysis and use	5		
**32. Standards for Community Health Volunteers**			
32. Disease prevention and environmental sanitation	1		
33. Home visiting	4		
34. Home management of minor ailments	2		
35. Community Outreaches	7		
36. Equipment, logistics and supplies	2		
**37. Standards for Community Health Committee**			
37. Governance, membership and operation	5		
38. Selection and supervision of community volunteers	2		
39. Welfare of CHO (include Security)	2		
40. Facility maintenance	2		
41. Resource mobilization and management	3		
**42. Standards for District Health Management Team (DHMT)**			
41. Management of CHPS Implementation	9		
42. Support to CHO	3		
43. Equipment	3		

Gaps

Performance standard Number	Gaps identified

ACTION PLAN

Gap Identified	Action to be taken	Person(s) responsible	Support needed	When will action be completed

Illnesses and Emergencies: 9) Infection Prevention 10) Malaria 11) TB 12) Hypertension 13) Diabetes 14) Sickel Cell Disease 15)Elephantiasis 16) Mental Health 17)Diarrhea 18) Worms 19)ARI 20)First Aid and Home Emergencies

9	Infection Prevention		Yes, No, N/A	Comments
9.1	**Supplies needed for infection prevention and control are available and ready to use**	**Verify by observation / interview that the following supplies are ready to use:**		
		Gauze/cotton wool is stored in dry and clean containers		
		Instruments are stored in dry and clean containers		
		Pick-up forceps are stored in dry and clean containers		
		Clean plastic containers with 0.5% chlorine solution are ready for use in all examination areas		
		Sharps containers which are less than 3/4 full are available in every consultation room		
9.2	**Provider correctly puts on sterile gloves**	**Verify by observation that the provider:**		
		Picks the right size of gloves		
		Washes and dries hands		
		Opens the sterile glove packet, putting the inner glove wrapper onto a clean surface with the cuffed ends facing towards the service provider		
		Opens the inner wrapper paper by touching the underside of the wrapper		
		Opens inner wrapper and pick up a glove with the hand the glove will not go on, grabbing it by the folded back cuff		
		Slips hand into the glove, only touching the interior aspect of the glove		
		Picks up the second glove with gloved hand by sliding fingertips under the sterile cuff of the second glove		
		Pulls the glove on while unfolding the cuff by pushing it up hand with fingers of the first gloved hand which are inside of the cuff		
9.3	**The decontamination of instruments is performed properly at site of use**	**Verify whether the provider correctly decontaminates equipment:**		
		Prepares a new chlorine solution at the beginning of each day or sooner		
		Disinfects equipment between patients, as per standard procedure		
		Uses chlorine diluted at 0.5%		
		Uses plastic containers for decontamination		
		Soaks instruments in 0.5% chlorine solution for 10 minutes before taken to the washing/prep room in a leak proof bucket		

**9.4**	**Cleaning/sterilizing of instruments is properly performed**	**Verify whether the person cleaning the instruments complies with the following steps and recommendations:**		
		There is a proper area for instrument processing / cleaning		
		Appropriate protective equipment is worn during the process: utility gloves, eyewear/face shield, plastic apron, enclosed shoes/gumboots		
		Scrubs instruments, completely removing all blood and other foreign matter with a soft brush, detergent, and chlorine solution		
		Rinses the instruments and other items thoroughly with clean water		
		Allows instruments and other items to air-dry OR dries with a clean towel (if autoclaving)		
		Washes hands with running water and soap for 10-15 seconds and dries		
**9.5**	**The sterilization process is performed properly**	**Verify whether high level disenfection processes are properly followed:**		
		All cleaned, disassembled instruments are totally immersed in water before lid is closed		
		Boils instruments for 20 minutes; counting from the time of boiling		
		Promptly removes instruments from boiling water with sterile forceps after 20 minutes and stores in HLD containers		
		Prepares the dressings for sterilzation, packing in a drum		
**9.6**	**Soiled linen is collected and cleaned properly**	**Verify whether the person cleaning the linens complies with the following steps and recommendations:**		
		Wears protective clothing: utility gloves, eye protection, enclosed shoes		
		Collects and transports soiled linen in leak proof containers/plastic bags		
		Cleans linen using detergents without acid, ammonia, or ammonium and mixed with chlorine bleach in the water		
		Washes hands with soap and water after removing gloves and other personal protective equipment		
**9.7**	**Waste is collected and disposed of properly to avoid injuries and contamination**	**Verify whether waste is collected properly:**		
		Wears protective clothing: utility gloves, eye protection, enclosed shoes		
		Collects waste in leak proof containers		
		Grounds (outside of the facility) are free of hospital waste		
		Person collecting waste washes hands with soap and water after removing gloves and other personal protective equipment		
		Sharps containers and solid waste are incinerated		
		Incineration area is not accessible to other staff, the community and domestic animals		
		Disposed waste should be burnt after each day		
**10**	**Malaria**			

10.1	**Provider appropriately welcomes and assesses the client suspected to have malaria**	**Verify by interviewing/observing that the provider:**		
		Builds rapport with the client / caregiver		
		Identifies **at least four common signs/symptoms of malaria:** fever, headaches, rigor (shivering), generalized body/joint pains, nausea/vomiting, loss of appetite, sweating, abdominal pain, diarrhoea, bitterness in mouth, irritability/refusal to feed (infants)		
		Checks temperature		
		Checks blood pressure		
		Checks weight		
		Checks heart rate		
		Checks respiratory rate		
		Records all findings in client folder		
		Checks for pallor of conjunctiva, lips and palms assessing for anemia		
		Checks for dryness of the lips, sunken fontanelles/eyes AND pinches the skin, assessing for dehydration		
10.2	**Diagnosis of malaria is made with a Rapid Diagnostic Test (RDT)**	**Verify by interviewing/observing that the provider:**		
		Checksthe expiry date of the malaria RDT, ensuring packaging is not damaged		
		Explains procedure to client		
		Washes hands and dries them		
		Swabs client's fingertip with alcohol swap, allowing it to dry		
		Hold figure firmly, pricking with lancer AND collecting sample of blood with pipette		
		Drops blood into second hole in the cassette followed by the correct amount of buffer solution		
		Reads and records results at time stipulated by RDT manufacturer		
		Informs clients of results of test		
		Discards kits (Observes infection preventions precautions throughout)		
10.3	**Clients diagnosed with malaria are provided appropriate care and treatment**	**Verify by interviewing/observing that the provider:**		
		Manages/describes how to manage high temperature in children: gives tepid sponge bath from feet to head AND gives tylenol suppository		

		Prescribes the correct dose of ACT according to weight, referring to a dosing table: Artesunate 4mg/Kg bodyweight (max 200mg) daily for 3 days; Amodiaquine 10mg/Kg bodyweight (max 600mg) OR Artemether Lumefantrine (according to dosing table for weight) twice daily for 3 days OR Dihydroartemisinin Piperaquine 1.6/12.8mg mg/kg daily for 3 days - to be taken after meals		
		Provider indicates that Artemether Lumefantine is NOT for children under 5kg or 6 months of age		
		Prior to administrating drugs to client, checks expiry date AND presence of AMFm green leaf sign on the packaging		
		Provides the patient/caregiver with information and education regarding the treatment side effects, advising to take pills after eating AND to ensure fluid intake		
		Advises client/caregiver that if vomiting occurs within 30 minutes after taking the medicine, dose of ACT should be repeated		
		Encourages client to return to health facility if no improvement after 24 hours OR symptoms worsen		
		Properly identifies/describes clients requiring referral: failure too respond to therapy OR severe disease (altered consciousness, repeated convulsion, inability to feed, severe anemia, jaundice)		
		Properly refers/describes referral process: educate patient on need for referral AND gives IM or rectal antimalarial prior to sending (quinine or Artemether) AND completes referral form		
**10.4**	**Health education is given on malaria prevention**	**Verify by interviewing/observing that the provider:**		
		Reviews the cause of malaria		
		Describes the effects of malaria during pregnancy		
		Emphasizes the importance/benefits of taking full course of SP during pregnancy AND completing treatment when diagnosed with malaria		
		Advises on use of LLIN every night AND **at least two other measures** to take to reduce risk of malaria transmission: wear protective clothing, use repellants, manage the environment, avoid being outside late at night, indoor residual spraying		
		Distributes/posts flyers/poster on malaria information		
**11**	**Tuberculosis**			
**11.1**	**People susepcted to have TB are**	**Verify by interview / observation that the provider:**		

	**identified and managed appropriately**	Identifies **at least four** common signs/symptoms of TB: cough for more than two weeks, weight loss, drenching night sweats, fever, loss of appetite, chest pain, haemoptysis		
		Asks if the client has had close contact with anyone diagnosed with TB		
		Checks weight		
		Checks temperature		
		Checks respiratory rate AND heart rate		
		Properly labels sputum container with client name, date of birth, and date of specimen collection		
		Educates the client how to properly collect sputum into the labeled container: rinse mouth first, take 3 deep breaths, and cough specimen into the container then immediately securing the lid		
		Requests the client to return the next day with an early morning sputum specimen (gives the patient a second labeled sputum container) as well as in 4 days to obtian results		
		Records that the specimen was collected in the Laboratory TB Investigation Form AND Case Identification AND Follow-up Regsiter prior to sending specimen to the lab		
11.2	**Clients diagnosed with TB are provided appropriate care and treatment**	**Determine by interviewing / observing that the provider:**		
		Determines TB treatment dosing of client according to weight		
		Educates the client about TB, **mentioning all of the following:** it is spread through the air by coughing; it is a disease of the lung but can affect other parts of the body; TB is curable		
		Educates the client on possible side effects of treatment, indicating when to seek care at the facility		
		Administers TB treatment to the client, ensuring proper number of tablets are provided AND informing client that treatment is free		
		Records dose(s) given on the patient treatment support card AND the TB client record		
		Provides support to the patient on treatment, assessing their health needs and providing support to ensure adherence		
		Traces clients in their home/community if they miss appointments		

		Encourages clients to cover the mouth with tissue or handkerchief when coughing AND always disinfect and wash it regularly		
		Offers the client testing and counseling for HIV		
12	**Hypertension**			
12.1	**Provider appropriately assesses and diagnoses the client with hypertension**	**Verify by observation / interview that the provider:**		
		Builds rapport with the client / caregiver		
		Checks blood pressure		
		Checks heart rate		
		Checks respiratory rate		
		Identifies hypertension if systolic BP more than 130 for three consecutive checks AND/OR diastolic BP more than 90 for three consectuive checks		
		Identifies **at least four common signs/symptoms** of hypertension: frequent headaches, tires easily, shortness of breath, weakness, dizziness, chest pain, asymptomatic		
12.2	**Clients diagnosed with hypertension are provided appropriate care and treatment**	**Verify by observation / interview that the provider:**		
		**Educates the client on at least three ways** to reduce risk: weight control; reduce alcohol consumption, decrease salt intake, rest, avoid stress, avoid smoking, undertake regular exercise, and reduce saturated fat in food		
		Informs clients why controlling blood pressure is important, **providing at least two examples** of possible effects: stroke, heart attack, congestive heart failure, enlarged heart		
		Refers newly diagnosed clients for treatment of hypertension		
		Routinely checks client's blood pressure at each visit, refering for further modification of therapy as necessary		
		Provides support to the patient on treatment, assessing their health needs and providing support to ensure adherence		
13	**Diabetes**			
13.1	**Provider appropriately assesses and diagnoses the client with diabetes**	**Verify by observation / interview that the provider:**		
		Builds rapport with the client / caregiver		
		Checks blood pressure		
		Checks heart rate		
		Checks respiratory rate		
		Checks weight		

		Identifies **at least four signs/symptoms** of diabetes: persistent presence of high sugar level in the blood and urine; continual thirst; frequent urination; tiredness; skin itching; recurrent infections; poor wound healing; weight loss; numbness in hands or feet		
		Indicates that diagnosis of diabetes requires a blood test to check glucose level in the blood		
13.2	**Clients diagnosed with diabetes are provided appropriate care and treatment**	**Verify by observation / interview that the provider:**		
		Educates the client on **at least three ways to reduce risk:** restrict intake of sugary food; eat foods low in starch; restrict alcohol intake; exercise regularly; eat a balanced diet consisting of fruits and vegetables;		
		Informs clients why controlling blood sugar is important, **providing at least two examples** of possible effects: stroke, loss of eye sight, kidney failure, poor circulation, sores on feet which may not heal		
		Refers clients suspecte of having diabetes / with diabetes for diagnosis / treatment		
		Provides support to the patient on treatment, assessing their health needs and providing support to ensure adherence		
14	Sickle Cell Disease			
14.1	**Provider appropriately assesses and diagnoses the client with sickle cell crises**	**Verify by observation / interview that the provider:**		
		Builds rapport with the client / caregiver		
		Checks blood pressure		
		Checks heart rate		
		Checks respiratory rate		
		Identifies **at least four common signs/symptoms** of sickle cell disease: joint pains; jaundice; abdominal pain; prominent forehead; thin/long hands and feet; small size for age		
		Identifies **at least two common signs/symptoms** of anemia: jaundice, weakness, pallor		
14.2	**Clients diagnosed with sickle cell disease are provided appropriate care and treatment**	**Verify by observation / interview that the provider:**		
		Refers clients in sickle cell crisis for pain management		
		Educates clients **on at least three ways to prevent sickle cell crisis:** drink plenty of fluids; maintain good nutrition; take folic acid daily; avoid cold temperatures; avoid high altitude; avoid overexposure of body parts		
		Encourages client to periodically check-up at a health facility		

15	**Elephantiasis / lymphatic filariasis**			
15.1	**Provider appropriately assesses and diagnoses the client with elephantiasis / lymphatic filariasis**	**Verify by observation / interview that the provider:**		
		Builds rapport with the client / caregiver		
		Checks blood pressure		
		Checks heart rate		
		Checks respiratory rate		
		Examines the skin		
		Identifies **at least two common signs/symptoms** of lymphatic filariasis: unusual swelling of legs, presence of skin folds, thickened skin,		
15.2	**Clients diagnosed with Elephantiasis / Lymphatic Filariasis are provided appropriate care and treatment**	**Verify by observation/ interview that the provider:**		
		Educates the client on the disease: that it is cause by a parasite transmitted by a mosquito that attacks the skin and the lymphatic system		
		Inform the client that it is alow disease, mostly affected in child hood but symptoms only show many years later		
		Educates client to sleep under mosquito nets at night and wear protective clothing to prevent transmission		
		Identifies correct treatment for the disease: Albendazole 400mg AND Ivermectin 150 – 200 mcg/kg or Diethylcarbamazine		
		Refers client for treatment, documenting the referral appropriately		
		Indicates importance of home visit to follow up clients with the disease AND to confirm outcome of referral		
16	**Mental Health**			
16.1	**Provider appropriately assessess and diagnoses the client with mental health / behavioural problems**	**Verify by interview / observation that the provider:**		
		Builds rapport with the client / caregiver		
		Checks blood pressure		
		Checks heart rate		
		Takes a health history of the client		
		Identifies **at least four signs/symptoms of** mental health problems in children: excessive crying; withdrawal; wetting the bed; restlessness; anti-social behavior; harming self; visual / auditory hallucinations; drug addiction; repeats tasks frequently (hand washing)		

		Identifies **at least four signs/symptoms of** mental health problems in adults: persistent insomnia; anxiety; palpitations; panic; excessive quietness; odd behavior; aggressive behavior; feels depressed; suicidal tendencies; weight gain; sexual dysfunction; intellectual deficiency		
**16.2**	**Clients with signs/symptoms or diagnosis of a mental illness are provided appropriate care and treatment**	**Verify by interview / observation that the provider:**		
		Appropriately refers the client with signs/symptoms of mental illness		
		Follows up the client to provide support, establishing rapport with the client and his/her family		
		Provides support to the patient on treatment, assessing their health needs and providing support to ensure adherence		
		Routinely counsels the client and his/her family		
		Records interaction with client in appropriate register		
**17**	**Diarrhea**			
**17.1**	**Provider appropriately assesses and diagnoses the client with diarrhea**	**Verify by observation / interview that the provider:**		
		Builds rapport with the client / caregiver		
		Checks blood pressure		
		Checks heart rate		
		Checks respiratory rate		
		Checks weight		
		Takes health history of client, identifying duration of diarrhea AND frequency and color of stools		
		Identifies **at least four common signs/symptoms of** dehydration: dry mouth, sunken eyes, sunken fontanels (children), loss of elasticity of skin, rapid weak pulse, lethargy, poor feeding, thirst		
**17.2**	**Clients with diarrhea are provided appropriate care and treatment**	**Verify by interview / observation that the provider:**		
		Educates clients on **at least three ways to prevent** diarrhea: avoid eating and drinking contaminated food and water; wash hands before eating; prepare food hygienically; store/cover food appropriately; clean feeding bottles; improve sanitation practices around the home; reheat left over food		
		Educates clients **on at least three ways to manage** diarrhea and dehydration: encourage to drink large amounts of fluids; give frequent sips of fluid; give fluids until diarrhea stops; slowly reintroduce foods; continue breastfeeding in infants		

		Gives zinc supplement daily for 10 days :10mg daily for children under 6 months of age OR 20mg daily for children over 6 months of age		
		Demonstrates how to prepare oral rehydration salts (ORS)		
		Encourages client to return to health facility if no improvement after 24 hours OR symptoms worsen		
		Properly identifies/describes clients requiring referral: failure to respond to therapy OR severe disease		
**18**	**Intestinal worms**			
**18.1**	**Provider appropriately assesses and diagnoses the client with intestinal worms**	**Verify by observation / interview that the provider:**		
		Builds rapport with the client / caregiver		
		Checks heart rate		
		Checks respiratory rate		
		Checks weight		
		Identifies **at least four common signs/symptoms** of intestinal worms: itching/irritation in the anus; weight loss/failure to thrive; nausea; weakness; constipation or diarrhea; abdominal pain; anaemia; dry cough; swollen abdomen; worm in stool/nose/mouth		
**18.2**	**Clients with intestinal worms are provided appropriate care and treatment**	**Verify by interview / observation that the provider:**		
		Educates the clients on **at least four ways** to prevent intestinal worms: use latrines; protect food from flies; cook meat well; wear footwear; periodic de-worming of children; provide good nutrition; ensure environmental hygiene		
		Provides appropriate treatment (Mebendazole or Albendazole according to protocol) for clients suspected to have intestinal worms		
		Educates clients on possible side effects of treatment (e.g. nausea, vomitting)		
		Properly identifies/describes clients requiring referral: failure to respond to therapy OR severe disease		
**19**	**Acute Respiratory Tract Infections**			
**19.1**	**Provider appropriately assesses and diagnoses the client with acute respiratory tract infection**	**Verify by interview / observation that the provider:**		
		Builds rapport with the client / caregiver		
		Check the temperature		
		Checks heart rate		
		Checks respiratory rate		
		Identifies **at least three common signs/ symptoms** of acute respiratory tract infection: cough, sore throat, running nose, difficulty breathing, restlessness		
**19.2**	**Clients with acute respiratory tract infections are provided appropriate care and treatment**	**Verify by interview / observation that the provider:**		
		Encourages client/caregiver to keep (child) well rehydrated		
		Administers paracetamol for fever		
		Provides amoxicillin according to weight as treatment		
		Educates client to eat a balanced diet		

		Encourages client to return to health facility if no improvement OR symptoms worsen		

		Properly identifies/describes clients requiring referral: failure to respond to therapy OR severe disease		
**20**	**First Aid and Home Emergencies**			
**20.1**	**Signs and symptoms of shock are recognized and managaed promptly**	**Verify by interview / observation that the provider:**		
		Identifies at least **four common signs/symptoms** of shock: fainting, restlessness, palpitations, pallor, sweating, confusion, coma, cold extremities, weak rapid pulse		
		Checks blood pressure		
		Checks heart rate		
		Checks respiratory rate		
		Checks temperature		
		Positions the patient flat on the back and turn head to one side, elevating the legs except in the case of head injury		
		Reassures and give warm fluids to conscious patients		
		Keeps client warm		
		Refers client for further investigation & treatment		
**20.2**	**Clients with animal bites are diagnosed and managed appropriately according to protocol**	**Verify by interview / observation that the provider:**		
		Builds rapport with the client / caregiver		
		Checks temperature		
		Assesses the wound and need for intervention		
		Irrigates/washs all cuts and scratches with soap and water		
		Gives the individual tetanus immunization		
		Reassures the client		
		Refers patient to the clinic for further care		
**20.3**	**Clients with snake bites are diagnosed and managed appropirately according to protocol**	**Verify by interview / observation that the provider:**		
		Builds rapport with the client / caregiver		
		Checks temperature		
		Washes the wound of poison, applying 'black stone' if available		
		Applies clean dressing, bandaging the affected part		
		Immobilizes the affected part		
		Gives anti-snake-venom if available		
		Reassures the client		
		Refers immediately to hospital for further management		
**20.4**	**Clients suspected of poisoning are**	**Verify by interview / observation that the provider:**		

	**diagnosed and managed appropriately according to protocol**	Identifies **at least four common signs/symptoms** of poisoning: nausea, vomiting, acute abdominal pain, diarrhea, faintness, shock, loss of consciousness		
		Takes a thorough history and identifies the poison as soon as possible		
		Checks blood pressure		
		Checks heart rate		
		Checks respiratory rate		
		Manages the poisoned client appropriately: administers an emitic to induce vomiting OR administers antidote OR administer aperient (caster oil/epsom salt) OR administers demulcent (milk/olive oil)		
		Reassures the client and refers as soon as possible		
		Refers client as appropriate		
**20.5**	**Signs and symptoms of convulsion/ seizures are identified and managed appropriately**	**Verify by interview / observation that the provider:**		
		Identifies **at least three common signs/symptoms** of seizures: violent movement of whole body or part of the body, tongue biting, foaming from the mouth, rolling of the eyes, unconsciousness		
		Checks heart rate		
		Prevents the patient from falling or harming themselves, lying them down with something under the head if possible and removing them from sources of danger		
		Reassures client and family members		
		Carefully and continually monitors the client's vital signs		
		Determines previous history of convulsion / seizure and any medication		
		For a child who is fitting and has a temperature >38 degrees C, immediately give 1 Paracetamol suppository		
		Refers immediately to hospital for management		
**20.6**	**Provider appropriately assesses and diagnosis the client with burns**	**Verify by interview / observation that the provider:**		
		Immediately secures the safety of the person		
		Take a health history from the client or caregiver, assessing the cause and severity of burn		
		Checks blood pressure		
		Checks heart rate		
		Classifies the severity of the burn: first degree (swollen and red) OR second degree (blistering) OR third degree (white/black in color, full-thickness burn		

		Assesses/clasifies the percentage of body that the burn covers: minor burn: all first and second degree burns that cover less than 10% of the body OR moderate and severe burns: involving more than 10% of the body surface area and all third degree involving more than 1%		
**20.7**	**Clients with burns are provided appropriate care and treatment**	**Verify by interview / observation that the provider:**		
		Removes burnt clothing and runs cool water over the affected area		
		Gently cleans and dries the injured area		
		Applies antibiotics such as Silver Sulphadiazine to mild and moderate burns OR do not apply anything to severe burns		
		Uses a sterile bandage to cover burns		
		Gives tetanus vaccination, if required		
		Makes patient comfortable, continuoulsy assesses breathing and pulse in those with severe burns		
		Ensure adequate hydration for mild and moderate burns		
		Refers clients with mild or moderate burns to the hospital		
**20.8**	**Sprains and strains are identified and managed appropriately**	**Verify by interview / observation that the provider:**		
		Builds rapport with the client / caregiver		
		Takes an appropriate health history regarding the injury		
		Identifies **at least three common signs/symptoms of** a sprain: severe pain increasing with movement of the affected joint; marked tenderness over the site of injury; swelling over site of injury; bruising; shock if in severe pain; loss of power of affected joint		
		Assesses the injury, determining range of motion and strength		
		Educates the client to rest the injured part but do not avoid all activity		
		Educates the client to ice the area using cold pack		
		Educates the client to compress the joint with an elastic wrap or bandage		
		Educates the client to elevate the injured part		
**20.9**	**Fractures and dislocation are identified, classified, and managed appropriately**	**Verify by interview / observation that the provider:**		
		Obtains a comprehensive history of the injury		
		Assessess the injured area for pain, tenderness, swelling, bruising, deformity, and ability to move		
		Assess circulation of affected limb or injured area		
		Classifies the injury appropriately: simple/closed fracture OR compound/open fracture OR dislocation		
		Covers any open wound immediately with clean dressing, stopping bleeding as necessary		
		Treats the client for shock if present		
		Immobilizes the part by splinting with bandage		
		Refers client immediately		
**20.10**	**Provider appropriately assesses and manages the client with epistasis**	**Verify by interview / observation the the provider:**		
		Identifies **at least two common causes of nose bleed:** trauma, infection, rhinitis (allergic and non allergic), hypertension		
		Takes a thorough health history, identifying possible causes of epistasis		

		Checks temperature		
		Checks blood pressure		
		Checks heart rate		
		Controls the bleeding by having the client sit and pinch the nose for at least ten minutes while leaning slightly forward		
		Applies cold pack / ice to nose and cheeks		
		Educates the client **on at least four factors to** prevent recurrent nose bleeds at home: rest with head elevated at 30 to 45 degrees; avoid putting objects in nose; avoid straining during bowel movements; avoid smoking; avoid medications that thin the blood (aspirin)		
		Properly identifies/describes clients requiring referral: failure to respond to therapy		
**20.11**	**Provider appropriately assesses and dresses wounds**	**Verify by observation / interview that the provider:**		
		Wash hands and dries them		
		Checks expiry date of dressing equipment and solution		
		Sets the tray with appropriate equipment to dress the wound		
		Explains the procedure to the client		
		Maintains clients privacy and comfort throughout the procedures		
		Administers analgesics as appropriate, allowing for time to take effect		
		Washes hands and dries them		
		Cleans the wound from inside to out with a series of swabs until clean and then appplies dressing to the cleaned wound		
		Discards used items, washes hands and document findings		

Community Linkages and Outreach: 21) Health Promotion and Education 22)Disease Survielence 23) Home Visits 24) School Health 25) Outreach 26) Health Volunteers 27) Community Health Committee

21	Health Promotion and Health Education		Yes, No, N/A	Comments
21.1	CHO prepares for a health education and health promotion by assessing community needs systematically	**Verify by interview / observation:**		
		A community profile		
		community map		
		demographic register		
		A community population		
21.2	Outcome of community walk around is analyzed	**Verify by observation/interview/records review of field reports captures the following:**		
		priority health issues (e.g. health condition or aminities such as public toilet, drinking water)		
		people affected		
		places where condition has been recorded		
		possible causes of the disease / lack of amenities		
21.3	Health education is prioritised and planned for jointly with CHC and CHV	**Determine by observation/interview/record review:**		
		Minutes of meeting held		
		findings from disease surveillance or community walk around are discussed		
		action plan developed		
		responsibilities are assigned		
		schedule developed for health education (target, topics, agenda)		
21.4	Health education is facilitated	**Verify by interview / observation:**		
		uses different communication mediums (e.g. dawn broadcase, community group, radio) appropriate for audience and topic		
		adapts approach for target audience (e.g. lanuage, terminology)		
		gives accurate information		

		determines understanding and addresses concerns and misconceptions		
		promotes participation, questions and discussion		
21.5	**Health education session is recorded and reported**	**Verify by interview / observation:**		
		Correct report format and register		
		Date, time, venue		
		Topics covered		
		Number of people participating		
		Follow up actions needed		
**22**	**Disease Surveillance**			
**22.1**	**Diseases requiring prompt reporting are identified**	**Verify by interview / observation:**		
		Cholera		
		Meningitis		
		Yellow Fever		
		Measles		
		Neonatal Tetanus		
		Acute Flaccid Paralysis		
		Guinea Worm Disease		
		Buruli Ulcer		
22.2	**Investigation of disease outbreaks are undertaken**	**Verify by interview / observation that the provider investigates:**		
		Compare previous trends and record		
		Who is getting the disease? (age, rich, poor, occupation)		
		What disease are they getting? (signs)		
		When did they start getting the disease? (season)		
		Where do the cases come from? (location)		
		Why are they getting the disease? (poor sanitation, lack of education)		
		How are they getting infected? (by drinking, bathing, wading in a particular water source)		
		Immediately take specimen from any case of flaccid paralysis		
22.3	**Community sub-structures are used for disease surveillance**	**Verify by interview / observation:**		
		TBAs and Traditional Healers		
		Chiefs and Elders		
		Unit Committee		
		Teachers or School Children		
		Community Health Committee		

		Area Council Members		
		Assembly men / women		
		Community Health Volunteers		
22.4	**Disease surveillance outbreaks are reported according to protocol**	**Verify by interview / observation:**		
		Correct forms are completed according to the type and severity of outbreak		
		Forms are submitted at the correct time and place		
23	**Home Visits**			
23.1	**Special clients are prioritized for home visits**	**Verify by interview / observation:**		
		Malnourished child		
		Buruli Ulcer patients		
		Aged		
		Orphans		
		Twin Mothers		
		Postnatal mother		
		HIV/AIDS patient		
		Grand multiparous		
		Physically and Mentally challenged people		
		Chronically ill patients (diabetic ,hypertensive, leprosy)		
		Preterm Babies		
23.2	**CHO prepares and adequately conducts home visits**	**Verify by interview / observation:**		
		Plans which area to go, arranging transport if necessary		
		Informs the CHV and CHCs at that community		
		Checks from registers if there are special clients to see in that community		
		Prepares home visiting bag		
		Maps out homes to be visited		
		Assesses the population dynamics (number of children to be immunized, number of pregnant women, etc…)		
		Sets the schedule wheel to indicate that Home Visits are being undertaken		
		Enters data on services provided during home visiting, reviewing action points		
24	**School Health**			
24.1	**CHO prepares and coordinates school health activities**	**Verify by interview / observation:**		
		Books appointment (date and time) with the school through the School Health Education Programme (SHEP) coordinator		
		Meets with the staff of the school especially the SHEP coordinator		
		Discuss the organization of the day's activities		
24.2	**Conducts health education session**	**Greets and introduces self to the students**		

	**for students**	Explains the purpose of the health education activity		
		Provides tailored information on the topic chosen		
		Speaks clearly and loudly and answers questions patiently and fully		
		Encourage pupils with individual problem to speak to the CHO after the session and assure confidentiality		
		Listen to, learn from and responds to the people in the group		
		Use audio visuals, job aids, teaching materials effectively		
**24.3**	**Physical examination of school children is conducted**	**Verify by interview / observation:**		
		Welcome child		
		Explain procedure		
		Wash hands with soap and running water before and at the end of the inspection		
		Use alcohol hand rub after inspecting each child		
		Inspect each child from head to toe for abnormalities , growths, discharges, swellings, parasitic infections e.g. lice		
		Observe for any abnormalities (including behaviour)		
		Document findings		
		Refer and hand over to SHEP coordinator		
**24.4**	**The physical environment of the school is inspected**	**Verify by interview / observation:**		
		State of school infrastructure		
		Sitting arrangement and capacity in a class		
		Ventilation in the classrooms		
		Availability and state of urinals and toilets		
		Refuse disposal sites		
		Recreational facilities or playground		
		Availability of trees to serve as wind breaks and shade for the pupils		
		Inspection of First Aid Box and its contents		
		Inspection of health status cards of food vendors ,the types of food sold, and cleanliness of the utensils and surroundings		
		Inspection of environmental certifacate		
		Findings recorded		
**24.5**	**A debriefing session is held with the relevant school authorities** [illegible]	**Verify by interview / observation:**		
		Number of children assessed including general findings, health status, nutrition and hygiene		

	**before the CHO leaves the school premises**	Numbers of children receiving services		
		Health education topics covered and number of children participating		
		Environmental findings and recommendations		
		Common health problems identified and actions that can be taken by the school		
		Review calendar for next visit and discuss any relevant preparations of issues to be addressed in the next visit		
**24.6**	**A report of the school visit and inspection is prepared and submitted**	**Verify by interview / observation:**		
		Name, date and time of visit to the school and SHEP Coordinator contacted		
		Total number of pupils enrolled in the school, number seen and number absent		
		Findings during the examination and inspection of the environment		
		Number of children assessed including general findings, health status, nutrition and hygiene		
		Numbers of children receiving services and referred		
		Health education topics covered and number of children participating		
		Recommendations		
		Action plan addressing health conditions and concerns		
		Set date of next visit		
**25**	**Outreach Activities**			
**25.1**	**CHO prepares for and conducts outreach services**	**Verify by observation / interview:**		
		Inform the CHC and CHV about the date of the outreach		
		Inform the CHC to beat the "gongong" to inform the communities about the outreach		
		Plan trip: arrange transport as needed AND pack equipment, drugs and supplies		
		Builds rapport with clients		
		Give a group education		
		Raises community awareness on importance of using ITNs		
		Community mental health sessions are conducted		
		Provide services: nutrition counselling, growth monitoring, immunization, family planning, ANC, management of minor ailments. (CHV is trained to assist here too)		
**25.2**	**After outreach activities are conducted appropriate records are made and follow up action is taken**	**Verify by observation/interview**		
		Tidies up the venue (in partnership with the CHV)		
		Pack her equipment and Logistics		
		Validates and Completes tallies/records for all services provided (in partnership with CHV)		
		Inform CHC and CHV about her departure		

**26**	**Supporting Community Health Volunteers**			
**26.1**	**Conducts monthly meeting with Community Health Volunteers**	**Verify by interview / observation:**		
		monthly meetings with CHC to find out activities undertaken in the community (Ask for minutes of meetings)		
		reviews Community Action Plan prepared by CHC (ask for Copy of Community Action Plan)		
		reviews achievements of Community Action Plan (ask for copies of decision taken as a result of evaluation done)		
		takes copies of records and reports submitted		
**27**	**Working with the Community Health Committee**			
**27.1**	**Preparation is undertaken for conducting a meeting with community health committee and community health volunteers**	**Verify by interview / observation:**		
		set date, time and venue of meeting with chairperson		
		prepare any documentation, data, action plan etc for disucssion		
		send reminders to committee		
**27.2**	**A community profile is completed for the communities served by the CHPS**	**Verify by interview / observation:**		
		Name of CHPS zone, including the community, including the villages that make up the community and the sub-district where it is located		
		Brief description of landscape and vegetation		
		Population of the zone or member villages		
		Main customs and beliefs of the people (religious groupings), including health seeking behavior		
		Economic activities: sources of income AND markets/shops that serve the community		
		Forms of transportation and communication		
		Housing-nature and pattern of housing, including access to water and sanitation facilities		
		Schools and other educational facilities		
		Health facilities (hospital, clinics, health Centre, community health compound, chemical shop, TBA compound)		
**27.3**	**The community served is mapped**	**Verify by interview / observation:**		
		Landmarks, land use, livestock		
		Water sources-rivers/streams		
		Schools, churches, mosques, shops, markets		
		Number and types of houses		
		Health and sanitation facilities		
		Chief's palace and meeting grounds		
		Streets/roads networks and other infrastructures		
		Police post, government office		
		Cemetery		
**27.4**	**Supports the activities of the Community Health Committee**	**Verify by interview / observation**		
		Serves as technical person in the monthly meetings of the CHC and CHVs		
		reviews minutes of the monthly meetings		

		Assist the CHC and CHVs to draw their work plan and implement it		
		Supervises and supports activities of CHC		

Management: 28) Planning 29) Logistics 30) Financial Management 31) Data Collection & Reporting

28	**Planning, implementing and managing activities**		Yes, No, N/A	Comments
28.1	**A monthly plan for activities is developed**	**Verify by observation/interviewing the CHO's monthly plan for activities to see that the following is included:**		
		Basic clinical services		
		Health education and outreach activities		
		Ordering and recieving drugs and supplies		
		Supervision		
		Data collection, reporting		
		Community Health Volunteers activities		
		Community Health Committee activities		
		Home visits		
28.2	**Demonstrates steps to follow in implementing planned activities**	**Verify by interviewing / observation: Discuss with SDHT , community health committee and CHV how to go about:**		
		Mobilize resources needed (e.g. IEC materials, contraceptives etc.)		
		Record all activities. Make observations and collect information using prepared tools		
		Arrange a follow up visit		
29	**Logistics Management**			
29.1	**Request for supplies follows the correct process**	**Verify by interview / observation**		
		Reporting, Requisitioning, Issuing and Receipt Voucher (RRIRV) completed		
		Reviewed and submitted to the regional medical stores every month (SDHT)		
		Complete the receipt portion of the RRIRV on receipt of supplies		
		File Waybill		
		Record supplies on appropriate bin cards		
29.2	**Supplies and equipment are managed**	**Verify by interview / observation:**		
		Keep up to date records of supplies by maintaining the bin cards, inventory and asset register		
		Order supplies and expendable materials using RRIRVs every month and conduct monthly stock taking		
		Maintain a maximum stock of 3 months for all supplies		
		stores drugs and supplies under right room temperature and conditions		

		issue drugs and supplies using the "First-to-Expire, First–Out"; and "First–In, First–Out" principle		
		provide adequate security for supplies (e.g. storeroom locked, bars on windows, fire safety)		
		Compile list of unserviceable items and expired drugs		
		Inform DHD and request collection / disposal		
**29.3**	**Adequate supplies of logistics are provided to the CHVs and TBAs to carry out their activities**	**Verify by interview / observation**		
		Meets CHVs monthly to take stock		
		Provide drugs and logistics to CHVs as needed		
**29.4**	**Vaccines are properly stored and maintained, adhering to cold chain requirements**	**Verify by observation / interview:**		
		Thermometer is placed inside the refrigerator AND/OR VVMs (Vaccine Vial Monitor) are used to properly to monitor temprature (inner is lighter than outer circle)		
		Tempurature read and documented on Temperature Chart twice daily (morning and evening)		
		Record receipt of vaccines onto ledgers and tally sheets		
		Keep vaccines **in the middle part** of the refridgerator between *2°C and *8°C (not on the door), while maintaining diluents in the bottom		
		Ice packs are packed in the freezer compartment at temperature less than 0°C to be used for Vaccine carriers and Vaccine cold box		
		FIFO system used, diposing of expired vaccines appropriately		
		Do not put food and drinks in the vaccine fridge		
		Do not open fridge door frequently		
**29.5**	**The CHPS compound is kept clean**	**Verify the absence of dust, cobwebs, blood, rubbish, used needles, swabs and syringes in the following areas:**		
		Consulting room		
		Instrument processing areas		
		Staff living area		
		Bathrooms		
		Drug storage area		
		EPI area		
		The area surrounding the facility		
**30**	**Financial Management**			
**30.1**	**Value Books are received, stored, and utilized appropriately**	**Verify by interviewing/observing the following**		
		Records value books serially in the stock register (used and unused)		
		Keep Value books safely and restrict access (used and unused)		
		Checks if pages are intact		
		Issues Value Books for use		

		Completes the appropriate columns in the stock register		
		Ensures the recipient signs against the entries in the register		
**30.2**	**Completed Value Books are received**	**Verify by interviewing/observing the following:**		
		Checks if pages are intact		
		Check if all leaflets are properly accounted for in the revenue collection cash book cash (in the case of GCRs)		
		Records details of Completed Value Books in appropriate columns of value Book Stock Register		
**30.3**	**Procurements utilize Value Books appropriately**	**Verify by interviewing/observing the following:**		
		Prepares requisition		
		Seeks approval for requisition from DHD		
		Submits approved requisition for supply to the Regional Medical Stores		
**30.4**	**Cash revenue is received and recorded according to protocol**	**Verify by interviewing/observing the following:**		
		Issues GCR to Payee		
		Records GCR in revenue Collection cash Book		
		Hands over daily cash Collected, GCR and Revenue Collection Cash Book		
		Acknowledges receipt of Daily Collections and paste receipts in the revenue collection book		
		Issues GCR to Payee		
**30.5**	**Revenue is banked and recorded**	**Verify by interviewing/observing the following:**		
		Prepares Bank pay-in slip in Triplicate		
		Pays Collections to the appropriate Bank Account at least weekly		
		Submits duplicate copy of Pay-in slip to the Sub-district/District finance Office for GCR		
		Keep triplicate of pay-in-slips		
		Maintains two Bank Accounts (Service and Drugs)		
**30.6**	**National Health Insurance claims are recorded and submitted**	**Verify by interviewing/observing the following:**		
		Records Daily Services to Clients (e.g. NHIS) and other cooperate bodies		
		Compiles Daily Claims		
		Submits Claims on a monthly basis		
**30.7**	**Cheques are collected and banked**	**Verify by interviewing/observing the following:**		
		Ensures Banking of Cheque Collections		
		Completes pay-in slip in triplicate		
		Records in Health Facilities(HF) IGF Service Analysis Cash Book and / or HF Drug Cash Book		
**30.8**	**Petty Cash is managed correctly**	**Verify by interviewing/observing the following:**		
		Establishes petty Cash Float (Imprest) from the DHA		
		Obtains approval of Petty Cash (Imprest)		
		Withdraws Petty Cash (Imprest)		
		Disburses Petty Cash (Imprest)		
		Records Petty Cash (Imprest) Payments		
		Recoups Petty Cash (Imprest) in petty cash book		

31	Data collection, reporting, analysis and use			
31.1	Data on all services provided are collected and recorded in the various registers	**Verify by observation / interview:**		
		Fills the entire applicable columns in the following registers correctly.		
		OPD register		
		Immunization register		
		Home visiting register		
		Antenatal Care register		
		Delivery Register		
		Post Natal Care Register		
		Consulting Room register (OPD)		
		Family Planning register		
31.2	Monthly Community-Based activities reported by CHCs and CHVs	**Verify by observation / interview:**		
		Home Visiting		
		Durbars		
		Malaria Case Management		
		Health Education		
		Environment Management		
		Intermittent Preventive Treatment (IPTp)		
31.3	CHPS data are analyzed and interpreted	**Verify by observation / interview:**		
		Displays monthly data graphically and / or tables of service delivery data at the CHPS zone (e.g. Immunization, Family Planning, ITN Use and Malaria Cases)		
		Calculates, plots and displays graphs showing coverage e.g. immunization, family planning and malaria cases		
		Compares data and trends overtime		
		Identifies gaps and priorities from the data		
31.4	CHO uses data for decision making	**Verify by observation / interview:**		
		Monthly facility meetings are held and data is discussed e.g. utilization, disease data, service coverage, or medicine stock outs are discussed		
		Decisions are made based on the data (Routine Health Information System) discussed		
		Action plans are developed to implement decisions		
		Meeting minutes are maintained		
31.5	CHPS data accuracy check is performed (If reported figure is higher than that of the register, then the data is over reporting and if the reported figure is less than what is on the register, then the data is under reporting)	**Complete the following information on the table below:**		
		In the first column, enter the data element of which you are assessing data quality		
		In the second column, enter the name of the service delivery form and its corresponding register where the selected data element can be found.		
		Randomly select two separate months reports		
		Enter the data at the appropriately at the ‘months’ cell and the figures reported under the report row.		

	Recount the figures in the register and write it down at the register column.		
	Writes remarks in the Comments column '***over reporting***' OR '***under reporting***'		
**Data Element Name**	**Source (***name of "reporting form" and "register"***)**	**a. Month:**	**b. Month:**
		**Report / Register**	**Report / Register**
1			
2			
3			
4			
5			

Equipment, tools, supplies, drugs

43	Necessary equipment and supplies are available	Yes / No	Comments
43.1	**Equipment**		
	Salter Weighing Scale		
	Blood Pressure Apparatus		
	Bowl to receive placenta		
	Couch		
	Demonstration tray, family planning		
	Fetoscope		
	Forceps, dressing		
	Galipots		
	Screen or curtain		
	Refrigerator		
	Refrigerator thermometer		
	Adult weighing Scale		
	Vaccine carrier		
	Clock/watch with second hand		
	**Logistics**		
	Cup with 100ml/50ml mark for tasting ORS solution (ORT)		
	Bucket, container for 0.5% chlorine		
	Bucket, container for contaminated waste		
	Mackintosh sheet		
	Benches and chairs at waiting area		
	Tape measure		
	Weighing pant		
	**Supplies**		
	Information, Education and Communication materials		
	Methylated Spirit		
	Alcohol Hand Rub		
	Plastic apron		
	Bandages / plaster		
	Chlorine solution or powder		
	Cotton swabs / gauze		

	Disposable syringes, 1 cc		
	Disposable syringes, 5 cc		
	Gloves, disposable		
	Gloves, sterile		
	Gloves, utility		
	Malaria Rapid Diagnostic Test		
	Scissors		
	Sheets, linen		
	Liquid soap		
	Thermometer		
	Waste containers: puncture proof for needles/syringes		
	Water, clean		
**43.2**	**Kits**		
	**Domiliary Midwifery kit**: plastic sheet, plastic apron, blade, cord ligatures, tablet of Misoprostol, cotton wool/gauze swabs, gloves, soap		
	**Sterile Delivery set**: sterile gauze/cotton wool, artery forceps, dipsosable gloves, umbilical clamp/sterile string, scissors, sponge holding forceps (2)		
**43.3**	**Monitoring and Evaluation tools**		
	Antenatal Care register		
	Child Health Record Book/weighing card		
	Consulting Room register (OPD)		
	Daily log		
	Delivery Register		
	Drug register		
	Drug Bin cards		
	Family Planning register		
	Family planning client record books		
	Family planning monthly reporting format		
	Immunization register		
	IMNCI chart booklet		
	Maternal Health Record Book		
	MCH quartery reporting format		
	Monthly Morbidity Report forms		
	Post Natal Care Register		

	Refrigerator temperature sheet		
	School Health Registration Notebook		
	TT Cards		
	Vaccine logistics register		
**44**	**Necessary medications are available**		
**44.1**	Albendazone		
	Amoxicillin		
	Artemether + lumefantrine		
	Artesunate + amodiaquine		
	Chloramphenicol eye ointment		
	Condoms		
	Cotrimoxazole		
	Depo-Provera (DMPA):		
	Distilled water		
	Ferrous folate		
	Gentian violet, 5%		
	Mebendazole tablet		
	Metronidazole		
	Misoprostol tablet		
	Multivitamin		
	Noristerat, Norigest (NET-EN):		
	Norygnon:		
	Oral Rehydration Solution and Zinc tablet		
	Oxcytocin, 10 units		
	Paracetamol		
	Sulfadoxine + Pyrimethamine		
	Tetanus Toxoid		
	Vitamin A		
