# Supplementary material for: Can a standards-based approach improve access to and quality of primary health care? Findings from an end-of-project evaluation in Ghana
Source: PLoS One. 2019 May 10;14(5):e0216589. doi: 10.1371/journal.pone.0216589 (PMC6510430; doi:10.1371/journal.pone.0216589)
Supplement: S2 Tool — (DOCX) [file pone.0216589.s003.docx]

**Household Survey Questionnaire – Tool 1**

Study Title: End-of-Project Evaluation of STAR-CHPS Project in Western Region, Ghana

IRB Number: 00006546

Version Number, Date: v2, 2015 October 20

Principal Investigator: Christina Maly

| **Date** |  | |  | |  | | |  | | 2015 | | | **Start Time** |  |  | : |  |  | 🞎 am  🞎 pm |
| --- | --- | --- | --- | --- | --- | --- | --- | --- | --- | --- | --- | --- | --- | --- | --- | --- | --- | --- | --- |
|  | Day | | | | Mo | | | | | Year | | |  |  |  |  |  |  |  |
| **Study ID** |  |  | |  | |  |  | |  | |  |  | **End Time** |  |  | : |  |  | 🞎 am  🞎 pm |
| **Reviewer** | | | | | | | | | | | | | **Data entry completed by** | | | | | | |
|  | | | | | | | | | | | | |  | | | | | | |
| **Signature** | | | | | | | | | | | | | **Signature** | | | | | | |
|  | | | | | | | | | | | | |  | | | | | | |

USE AFTER SCREENING AND CONSENTING CLIENT

Thank you again for agreeing to participate in this interview. Let me tell you a little about the process. I will ask you a few questions about yourself, your experiences with seeking health care, and general health knowledge. In some cases, I will be asking you to answer questions in your own words. For other questions, you will be given a set of answers and asked to choose the one that is closest to your own view. Even though none of the answers may fit your ideas exactly, choosing the answer closest to your views will help us to compare your answers more easily with those of other people. It is very important that you answer as accurately as you can. Take your time, and ask me to clarify if you have any question about what is wanted. Please keep in mind all information you provide will be confidential and anonymous. You may choose to not answer any question, and you can stop the interview at any time.

1. (DO NOT READ. CODE SEX)

Male 0

Female 1

Not known 98

2. First I’d like to ask you a few questions about yourself. What is your age?

|  |  |
| --- | --- |

Don’t know (END) 98

No response (END) 99

IF RESPONDENT IS LESS THAN 18, END INTERVIEW

3. What is your current marital status? Would you say. . .

You are married or living together, 1

You are divorced separated, 2

Widowed, 3

Or have you never been married or lived together? 4

Don’t know 98

No response 99

4a. How many living children do you have?

0 0

1 1

2 2

3 3

4 4

5 5

More than 5 6

Don’t know 98

No response 99

4b. IF >5 SPECIFY

|  |  |
| --- | --- |

Don’t know 98

No response 99

5. Have you ever attended school or received nonformal education? Or neither?

Neither (GO TO Q7) 0

Nonformal education (GO TO Q7) 1

Attended School 2

Don’t know (GO TO Q7) 98

No response (GO TO Q7) 99

6. What is the highest level of school you attended?

Primary school 1

Junior high school (JHS)/JSS/Middle School 2

Senior high school (SHS)/SSS/Technical/Vocational 3

Tertiary/Higher 4

Not applicable 97

Don’t know 98

No response 99

7. What is the primary way you make a living?

Fisher/Fishmonger 1

Petty trader 2

Farmer 3

Civil / Public servant 4

Student 6

Unemployed 7

Retired 8

Other, specify: __________________ 96

Don’t know 98

No response 99

8. What is your religion? (PROBE: What is your denomination?)

Anglican 1

Catholic 2

Methodist 3

Pentecostal/Charismatic 4

Presbyterian 5

Other Christian 6

Traditional/spiritualist 7

Muslim 8

No religion 9

Other 96

Don’t know 98

No response 99

10. Thinking of the toilet facility you and your family usually use, do you share this toilet facility?

No 0

Yes 1

Don’t know 98

No response 99

11. Now, I’d like to ask you about some items people in this community may or may not have in their homes. As for your home, please tell me if you have the following items. Do you have ... (READ A-FG)?

|  | Yes | No | Don’t know | No response |
| --- | --- | --- | --- | --- |
| 1. Electricity? | 1 | 0 | 98 | 99 |
| 1. A radio? | 1 | 0 | 98 | 99 |
| 1. A television? | 1 | 0 | 98 | 99 |
| 1. A bicycle? | 1 | 0 | 98 | 99 |
| 1. A mobile phone? | 1 | 0 | 98 | 99 |
| 1. A motorbike? | 1 | 0 | 98 | 99 |

12. Would you describe your health as . . .

Excellent, 1

Very good, 2

Fair, 3

Or poor? 4

Don’t know 98

No response 99

13a. Do you have a national health insurance registration card?

No (GO TO Q14) 0

Yes 1

Don’t know 98

No response 99

13b. Is the card active? (PROBE: Is the card current, or has it expired?)

No/Expired 0

Yes/Active 1

Not applicable 97

Don’t know 98

No response 99

14. Now I’d like to speak with you about the health services available in your area. Thinking over the last year, please tell me where you have gone, or taken a family member, to receive health care. (DO NOT READ ANSWERS. PROBE: Anywhere else?)

|  | Mention | Not Mention | Don’t know | No response |
| --- | --- | --- | --- | --- |
| 1. CHPS compound | 1 | 0 | 98 | 99 |
| 1. Health center | 1 | 0 | 98 | 99 |
| 1. District hospital | 1 | 0 | 98 | 99 |
| 1. Private provider / maternity clinic | 1 | 0 | 98 | 99 |
| 1. Drug shop / chemical seller / chemist | 1 | 0 | 98 | 99 |
| 1. Traditional healer / shrine owner / Herbalists / Atheists | 1 | 0 | 98 | 99 |
| 1. Traditional birth attendant | 1 | 0 | 98 | 99 |
| 1. Other, specify: ______________ | 1 | 0 | 98 | 99 |

15. Now, I’d like to speak with you specifically about your experience with the nearest community health facility, also known as [CHPS ZONE NAME]. When was the last time you went to [CHPS ZONE NAME] either for your own health, or for that of a family member?

Never 0

Less than a month (GO TO Q17) 1

1-2 months (GO TO Q17) 2

3-6 months (GO TO Q17) 3

6-12 months (GO TO Q17) 4

More than a year ago (GO TO Q17) 5

Don’t know (GO TO Q17) 98

No response (GO TO Q17) 99

16a. Tell me the reasons you have never visited [CHPS ZONE NAME].

(DO NOT READ ANSWERS. PROBE: Any other reason?)

*Don’t know” or “No response” cannot be selected if any other answer choices are mentioned*

|  |  | Mention | Not Mention | Don’t know | No response |
| --- | --- | --- | --- | --- | --- |
| **PHYSICAL** | |  |  |  |  |
|  | Too far / distance | 1 | 0 | 98 | 99 |
|  | Provider not available when needed | 1 | 0 | 98 | 99 |
| **FINANCIAL** | |  |  |  |  |
|  | Cannot afford transportation | 1 | 0 | 98 | 99 |
|  | Cannot afford services / drugs | 1 | 0 | 98 | 99 |
|  | Cannot afford to not work | 1 | 0 | 98 | 99 |
| **QUALITY** | |  |  |  |  |
|  | Wait time | 1 | 0 | 98 | 99 |
|  | Lack of privacy | 1 | 0 | 98 | 99 |
|  | Providers are NOT competent | 1 | 0 | 98 | 99 |
|  | Poor treatment by providers | 1 | 0 | 98 | 99 |
|  | Spouse/family prefers me not to | 1 | 0 | 98 | 99 |
| **ACCEPTABILITY** | |  |  |  |  |
|  | Receive health care elsewhere | 1 | 0 | 98 | 99 |
|  | Does not provide needed services | 1 | 0 | 98 | 99 |
|  | Other, specify: ______________ | 1 | 0 | 98 | 99 |

Not applicable 97

ALL RESPONSES GO TO Q33

17a. How many times have you visited [CHPS ZONE NAME] in the last year, either for yourself or with a member of your household?

Never (GO TO Q33) 0

1 1

2 2

3 3

4 4

5 5

6 6

7 7

8 8

9 9

More than 9 10

Not applicable (GO TO Q33) 97

Don’t know (GO TO Q33) 98

No response (GO TO Q33) 99

17b. IF >9, SPECIFY

|  |  |
| --- | --- |

Not applicable 97

Don’t know 98

No response 99

18. Now I would like to ask you several questions about your most recent visit to [CHPS ZONE NAME]. At the time of your most recent visit, who was seeking care? (DO NOT READ ANSWERS. PROBE: Anyone else?)

|  |  | Mention | Not Mention | Don’t know | No response |
| --- | --- | --- | --- | --- | --- |
|  | Yourself | 1 | 0 | 98 | 99 |
|  | Child in your household | 1 | 0 | 98 | 99 |
|  | Other adult in your household | 1 | 0 | 98 | 99 |
|  | Family member outside household | 1 | 0 | 98 | 99 |
|  | Friend | 1 | 0 | 98 | 99 |
|  | Other, specify: ______________ | 1 | 0 | 98 | 99 |

Not applicable 97

19. What was the reason for this visit? (DO NOT READ ANSWERS. PROBE: Anything else?)

|  |  | Mention | Not Mention | Don’t know | No response |
| --- | --- | --- | --- | --- | --- |
| **CHILD HEALTH** | |  |  |  |  |
|  | Immunization | 1 | 0 | 98 | 99 |
|  | Nutrition / Breastfeeding / Diet | 1 | 0 | 98 | 99 |
|  | Acute care/ Illness (diarrhea, upper respiratory) | 1 | 0 | 98 | 99 |
| **REPRODUCTIVE HEALTH** | |  |  |  |  |
|  | Family planning | 1 | 0 | 98 | 99 |
|  | HIV/AIDS/ Sexually transmitted infections | 1 | 0 | 98 | 99 |
|  | Antenatal care | 1 | 0 | 98 | 99 |
|  | Labor and delivery | 1 | 0 | 98 | 99 |
|  | Postnatal and newborn care | 1 | 0 | 98 | 99 |
| **ILLNESS** | |  |  |  |  |
|  | Infectious diseases (malaria, TB, measles, cholera) | 1 | 0 | 98 | 99 |
|  | Mental health | 1 | 0 | 98 | 99 |
|  | First aid | 1 | 0 | 98 | 99 |
|  | Hypertension / Diabetes | 1 | 0 | 98 | 99 |
|  | Other, specify: ______________ | 1 | 0 | 98 | 99 |

Not applicable 97

20. In your most recent visit, what was the primary way you got to [CHPS ZONE NAME]?

By foot 1

By bicycle 2

By motorcycle 3

By car 4

By bus 5

Not applicable 97

Don’t know 98

No response 99

21. In your most recent visit, how long did it take?

Less than 15 minutes 1

15-29 minutes 2

30-59 minutes 3

1-2 hours 4

More than 2 hours 5

Not applicable 97

Don’t know 98

No response 99

22. How do you see the time it took to get from your home to [CHPS ZONE NAME]? Would you say it was …

Reasonable, 1

Somewhat reasonable, 2

Or not at all reasonable? 3

Not applicable 97

Don’t know 98

No response 99

23. At this visit, how did you feel about the amount of time the CHPS health care provider spent with you? Would you say it was . . .

Too long, 1

Not enough time, 2

Or just the right amount of time? 3

Not applicable 97

Don’t know 98

No response 99

24. Based on your last visit to [CHPS ZONE NAME], would you say the CHPS health care provider was . . .

Very respectful, 1

Somewhat respectful, 2

Or not at all respectful? 3

Not applicable 97

Don’t know 98

No response 99

25. Thinking about your most recent visit, would you agree or disagree with this statement: I can trust that the CHPS health care provider will keep my health and personal information private. Would you …

Strongly agree, 1

Somewhat agree, 2

Somewhat disagree, 3

Or strongly disagree? 4

Not applicable 97

Don’t know 98

No response 99

26. Thinking about your most recent visit, would you agree or disagree with this statement: I believe that the CHPS health care provider has the knowledge and skills to help me with my health needs. Would you …

Strongly agree, 1

Somewhat agree, 2

Somewhat disagree, 3

Or strongly disagree? 4

Not applicable 97

Don’t know 98

No response 99

27. Thinking about your most recent visit to [CHPS ZONE NAME], how much of a problem was privacy? That is, having others see or hear you. Would you say this was …

A major problem, 1

A minor problem, 2

Or not a problem at all? 3

Not applicable 97

Don’t know 98

No response 99

28. Now, thinking about the amount of time you had to wait to see a CHPS health care provider at your most recent visit to [CHPS ZONE NAME], would you say the amount of time you waited was…

Reasonable, 1

Somewhat reasonable, 2

Or not at all reasonable? 3

Not applicable 97

Don’t know 98

No response 99

29. During your last visit, did the CHPS health care provider recommend you take some drugs or medications?

No (GO TO Q31) 0

Yes 1

Not applicable (GO TO Q31) 97

Don’t know (GO TO Q31) 98

No response (GO TO Q31) 99

30. At that visit, were you able to get all of the drugs that the CHPS health care provider recommended, some of the drugs recommended, or were you not able to get any of the drugs at [CHPS ZONE NAME]?

None 0

Some 1

All 2

Didn’t need drugs 3

Not applicable 97

Don’t know 98

No response 99

31. Did you have to pay money, for any reason, at your last visit to [CHPS ZONE NAME]?

No (GO TO Q33) 0

Yes 1

Don’t know (GO TO Q33) 98

No response (GO TO Q33) 99

32. How affordable were those fees? Would you say they were . . .

Very affordable, 1

Somewhat affordable, 2

Somewhat unaffordable, 3

Or very unaffordable? 4

Not applicable 97

Don’t know 98

No response 99

33. If you, or a member of your family, was exhibiting signs of malaria, how likely are you to go to [CHPS ZONE NAME] or see a CHPS health care provider? Are you …

Very likely, 1

Somewhat likely, 2

Or not at all likely? 3

Don’t know 98

No response 99

34. If you or a member of your household wanted to space or prevent pregnancies, how likely are you to go to [CHPS ZONE NAME] or see a CHPS health care provider? Would you be …

Very likely, 1

Somewhat likely, 2

Or not at all likely? 3

Don’t know 98

No response 99

35. If a child in the household needed to be immunized, how likely are you to go to [CHPS ZONE NAME] or see a CHPS health care provider? Are you …

Very likely, 1

Somewhat likely, 2

Or not at all likely? 3

Don’t know 98

No response 99

36. Overall thinking of the care you, or members of your household, have received from [CHPS ZONE NAME] health care providers, how satisfied are you? Would you say you are …

Very satisfied, 1

Somewhat satisfied, 2

Somewhat dissatisfied, 3

Or very dissatisfied? 4

Don’t know 98

No response 99

37a. As you may know, both CHPS health care providers and other members of the community visit people’s homes to talk about health or provide services. For now, I’d like you to think only about the CHPS health care providers, who people sometimes call “nurse” or “doctor.”

Thinking over the last year, how many times has a CHPS health care provider visited your home?

0 (GO TO Q45a) 0

1 1

2 2

3 3

4 4

More than 4 5

Not applicable 97

Don’t know 98

No response 99

37b. IF >4, SPECIFY

|  |  |
| --- | --- |

Don’t know 98

No response 99

38a. Did you participate in any of those visits made by the CHPS health care provider?

No (GO TO Q45a) 0

Yes 1

Not applicable 97

Don’t know 98

No response 99

38b. When was the last time you participated in any of those visits made by a CHPS health care provider made to your home?

Never (GO TO Q45a) 0

Less than a week 1

1 week - less than a month 2

1-3 months 3

4-6 months 4

7-12 months 5

More than a year ago 6

Not applicable 97

Don’t know 98

No response 99

39. Now I’d like to ask you a few questions about the last time a CHPS health care provider visited your home. During that most recent visit, what services did the CHPS health care provider provide? (DO NOT READ ANSWERS. PROBE: Anything else?]

|  |  | Mention | Not Mention | Don’t know | No response |
| --- | --- | --- | --- | --- | --- |
| **CHILD HEALTH** | |  |  |  |  |
|  | Immunization | 1 | 0 | 98 | 99 |
|  | Nutrition | 1 | 0 | 98 | 99 |
|  | Acute care/ Illness (diarrhea, upper respiratory) | 1 | 0 | 98 | 99 |
| **REPRODUCTIVE HEALTH** | |  |  |  |  |
|  | Family Planning | 1 | 0 | 98 | 99 |
|  | HIV / AIDS and sexually transmitted infections | 1 | 0 | 98 | 99 |
|  | Antenatal Care | 1 | 0 | 98 | 99 |
|  | Postnatal & Newborn care | 1 | 0 | 98 | 99 |
| **ILLNESS** | |  |  |  |  |
|  | Infectious Diseases (malaria, TB, measles, cholera, etc) | 1 | 0 | 98 | 99 |
|  | Mental Health | 1 | 0 | 98 | 99 |
|  | First Aid | 1 | 0 | 98 | 99 |
|  | Hypertension & Diabetes | 1 | 0 | 98 | 99 |
|  | Health education |  |  | 98 | 99 |
|  | Other, specify:___________ | 1 | 0 | 98 | 99 |

Not Applicable 97

40. Thinking about the length of time the CHPS health care provider spent at your home during this most recent visit, was it . . .

Too long, 1

Too short, 2

Or just the right amount of time? 3

Not applicable 97

Don’t know 98

No response 99

41. I am going to read you a few statements about CHPS health care providers visiting your home. For each statement tell me how much you agree.

I am glad when a CHPS health care provider visits my home.

How much do you agree with this statement? Would you say you …

Strongly agree, 1

Somewhat agree, 2

Somewhat disagree, 3

Or do you strongly disagree? 4

Not applicable 97

Don’t know 98

No response 99

42. The CHPS health care provider is respectful when visiting my home. Would you say you...

Strongly agree, 1

Somewhat agree, 2

Somewhat disagree, 3

Or do you strongly disagree? 4

Not Applicable 97

Don’t know 98

No response 99

43. Thinking about the most recent visit at your home, would you agree or disagree with this statement: I can trust that the CHPS health care provider will keep my health and personal information private. Would you ...

Strongly agree, 1

Somewhat agree, 2

Somewhat disagree, 3

Or do you strongly disagree? 4

Not Applicable 97

Don’t know 98

No response 99

44. Thinking about your most recent visit at your home, would you agree or disagree with this statement: I believe that the CHPS health care provider has the knowledge and skills to help me with my health needs. Would you ...

Strongly agree, 1

Somewhat agree, 2

Somewhat disagree, 3

Or do you strongly disagree? 4

Not Applicable 97

Don’t know 98

No response 99

45a. As you may know, there are also volunteers in this community who visit homes to discuss health and sanitation with community members. These people are not doctors or nurses.

How many times has a community health volunteer visited your home in the past year, or has one not visited?

0 (GO TO Q53) 0

1 1

2 2

3 3

4 4

More than 4 5

Not applicable 97

Don’t know 98

No response 99

45b. IF >4, SPECIFY

|  |  |
| --- | --- |

46a. Did you participate in any of those visits made by the community health volunteer?

No (GO TO Q52) 0

Yes 1

Not applicable 97

Don’t know 98

No response 99

46b. When was the last time you participated in any of those visits made by the community health volunteer?

Never (GO TO Q49) 0

Less than a week 1

1 week - less than a month 2

1-3 months 3

4-6 months 4

7-12 months 5

More than a year ago (GO TO Q49) 6

Not applicable 97

Don’t know (GO TO Q49) 98

No response (GO TO Q49) 99

47. Thinking about the length of time the community health volunteer spent at your home during this most recent visit, was it . . .

Too long, 1

Too short, 2

Or just the right amount of time? 3

Not applicable 97

Don’t know 98

No response 99

48. Thinking about the most recent visit at your home, would you agree or disagree with this statement: I believe that the community health volunteer has the knowledge and skills to help me with my health needs. Would you say you ...

Strongly agree, 1

Somewhat agree, 2

Somewhat disagree, 3

Or do you strongly disagree? 4

Not Applicable 97

Don’t know 98

No response 99

49. I am going to read you a few statements about community health volunteers visiting your home. For each statement tell me how much you agree.

I am glad when a community health volunteer visits my home. Would you say you …

Strongly agree, 1

Somewhat agree, 2

Somewhat disagree, 3

Or do you strongly disagree? 4

Not Applicable 97

Don’t know 98

No response 99

50. When I receive drugs from the community health volunteer, I trust they are safe and effective. Would you say you …

Strongly agree, 1

Somewhat agree, 2

Somewhat disagree, 3

Or do you strongly disagree? 4

Not Applicable 97

Don’t know 98

No response 99

51. How much do you agree with this statement? The community health volunteer is respectful when visiting my home. Would you say you...

Strongly agree, 1

Somewhat agree, 2

Somewhat disagree, 3

Or do you strongly disagree? 4

Not Applicable 97

Don’t know 98

No response 99

52. I would like to ask you about a couple of types of events that may, or may not happen in your community. Thinking over the last year, have you attended a durbar where a CHPS health care provider has given a health talk?

No 0

Yes 1

Don’t know 98

No response 99

IF RESPONDENT HAS NO CHILDREN (SEE Q4a), GO TO Q54

53. Thinking again about the past year, have you attended a child welfare clinic, which some people call “weighing,” either in the community or at the [CHPS ZONE NAME]?

No 0

Yes 1

Don’t know 98

No response 99

54. Now, I would like to ask you a few questions that you may, or may not know the answers to. If you do not know, that is okay, just let me know.

From what you know or have heard, how can a person get malaria, or are you unsure? (DO NOT READ ANSWERS. PROBE: Anything else?)

|  |  | Mention | Not Mention | Don’t know | No Response |
| --- | --- | --- | --- | --- | --- |
|  | Mosquito | 1 |  | 98 | 99 |
|  | Fly | 1 | 0 | 98 | 99 |
|  | Standing in the sun | 1 | 0 | 98 | 99 |
|  | Eating green vegetables | 1 | 0 | 98 | 99 |
|  | Eating starchy food | 1 | 0 | 98 | 99 |
|  | Witchcraft | 1 | 0 | 98 | 99 |
|  | Working too hard | 1 | 0 | 98 | 99 |

Other 96

55. What are the common signs and symptoms of malaria, or are you unsure? (DO NOT READ ANSWERS. PROBE: Anything else?)

|  | Mention | Not Mention | Don’t know | No Response |
| --- | --- | --- | --- | --- |
| 1. High temperature/Fever | 1 | 0 | 98 | 99 |
| 1. Loss of energy | 1 | 0 | 98 | 99 |
| 1. Vomiting | 1 | 0 | 98 | 99 |
| 1. Sweating | 1 | 0 | 98 | 99 |
| 1. Headache | 1 | 0 | 98 | 99 |
| 1. Body pains | 1 | 0 | 98 | 99 |
| 1. Itching | 1 | 0 | 98 | 99 |
| 1. Loss of Appetite | 1 | 0 | 98 | 99 |
| 1. Chills | 1 | 0 | 98 | 99 |
| 1. Dizziness | 1 | 0 | 98 | 99 |
| 1. Other, specify:_________ | 1 | 0 | 98 | 99 |

56. Now, I’d like to ask you about birth spacing, that is, having some time in between one pregnancy and the next. From what you know or have heard, are there health benefits to birth spacing for **babies**, or are you unsure?

No 0

Yes 1

Don’t know 98

No response 99

57. From what you know or have heard, are there health benefits to birth spacing for **mothers,** or are you unsure?

No 0

Yes 1

Don’t know 98

No response 99

58. How about immunization? Are there health benefits to having a child vaccinated?

No (END) 0

Yes 1

Don’t know (END) 98

No response (END) 99

59. What are the health benefits to having a child vaccinated? (DO NOT READ ANSWERS. PROBE: Anything else?)

*Don’t know” or “No response” cannot be selected if any other answer choices are mentioned.*

|  | Mention | Not Mention | Don’t know | No response |
| --- | --- | --- | --- | --- |
| 1. Prevent my child from getting sick | 1 | 0 | 98 | 99 |
| 1. Prevent other members in the household from getting sick | 1 | 0 | 98 | 99 |
| 1. Lessen disease severity | 1 | 0 | 98 | 99 |
| 1. Keeps community healthy | 1 | 0 | 98 | 99 |
| 1. Other, specify: _____________ | 1 | 0 | 98 | 99 |

Not applicable 97

Thank you again for taking time to do this survey. As a token of our appreciation, please accept this bar of soap and detergent. We value your participation. Have a good day.

60. (DO NOT READ. RECORD MAIN MATERIAL OF ROOF.)

Natural/Rudimentary 1

Finished 2

Don’t know 98

**NATURAL/RUDIMENTARY ROOFING**

- Palm Leaf/Raffia
- Rustic mat
- Bamboo
- Wood Planks
- Cardboard

**FINISHED ROOFING**

- Metal
- Wood
- Calamine/cement fiber
- Ceramic / Brick tiles
- Cement
- Roofing shingles
- Asbestos/Slate roofing sheets

61. (DO NOT READ. RECORD MAIN MATERIAL OF WALLS.)

Natural/Rudimentary 1

Finished 2

Don’t know 98

**NATURAL /RUDIMENTARY WALLS**

- Cane/Palm/Trunks
- Dirt
- Bamboo with mud
- Stone with mud
- Uncovered adobe
- Plywood
- Cardboard
- Reused wood

**FINISHED WALLS**

- Cement
- Stone with lime/cement
- Bricks
- Cement blocks
- Covered adobe
- Woodplank/shingles

62. (DO NOT READ. RECORD MAIN MATERIAL OF FLOOR.)

Natural 1

Rudimentary 2

Finished 3

Don’t know 98

**NATURAL FLOOR**

- Earth/Sand
- Dung

**RUDIMENTARY FLOOR**

- Wood planks
- Palm/bamboo

**FINISHED FLOOR**

- Parquet or polished wood
- Vinyl or asphalt strips
- Ceramic tiles
- Cement
- Carpet
